# Tetrameric c-di-GMP Mediates Effective Transcription Factor Dimerization to Control *Streptomyces* Development

**DOI:** 10.1016/j.cell.2014.07.022

**Published:** 2014-08-28

**Authors:** Natalia Tschowri, Maria A. Schumacher, Susan Schlimpert, Naga babu Chinnam, Kim C. Findlay, Richard G. Brennan, Mark J. Buttner

**Affiliations:** 1 Department of Molecular Microbiology, John Innes Centre, Norwich Research Park, Norwich NR4 7UH, UK; 2 Department of Biochemistry, Duke University School of Medicine, Durham, NC 27710, USA

## Abstract

The cyclic dinucleotide c-di-GMP is a signaling molecule with diverse functions in cellular physiology. Here, we report that c-di-GMP can assemble into a tetramer that mediates the effective dimerization of a transcription factor, BldD, which controls the progression of multicellular differentiation in sporulating actinomycete bacteria. BldD represses expression of sporulation genes during vegetative growth in a manner that depends on c-di-GMP-mediated dimerization. Structural and biochemical analyses show that tetrameric c-di-GMP links two subunits of BldD through their C-terminal domains, which are otherwise separated by ∼10 Å and thus cannot effect dimerization directly. Binding of the c-di-GMP tetramer by BldD is selective and requires a bipartite RXD-X_8_-RXXD signature. The findings indicate a unique mechanism of protein dimerization and the ability of nucleotide signaling molecules to assume alternative oligomeric states to effect different functions.

## Highlights

• c-di-GMP controls development in the multicellular bacteria *Streptomyces*
• c-di-GMP developmental signaling is directly mediated by the master regulator BldD
• A heretofore unseen tetrameric form of c-di-GMP binds BldD to effect its dimerization
• BldD-(c-di-GMP) represses transcription of sporulation genes during vegetative growth

## Graphical Abstract

**Figure:**
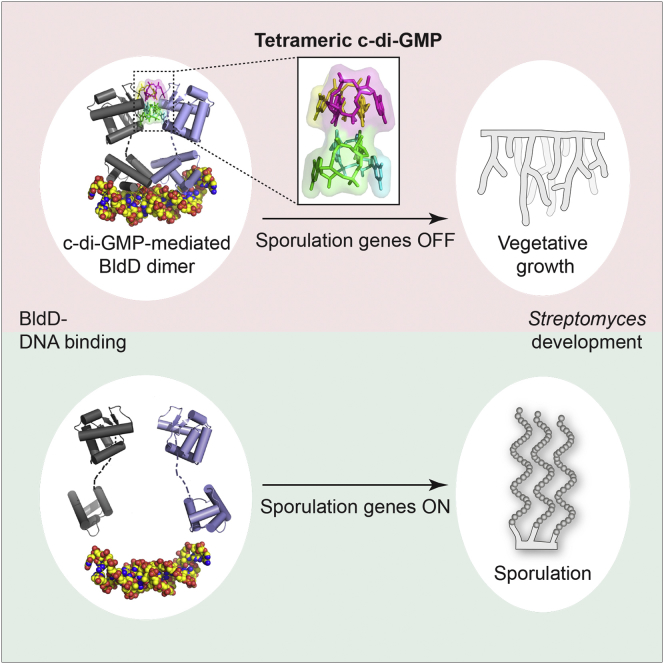

## Teaser

A ci-d-GMP tetramer functions as molecular glue for dimerization of a transcription factor leading to its activation, which is important for developmental transitions in a sporulating bacterium.

## Introduction

In all domains of life, nucleotide-based second messengers allow a rapid integration of external and internal signals into fine-tuned regulatory pathways that control cellular responses to changing conditions. As a unifying theme, a basic second messenger control module consists of two distinct enzymes for synthesis and degradation of the second messenger and a nucleotide sensor that, upon ligand binding, interacts with a target to produce a cellular output (Hengge, 2009). 3′, 5′-cyclic diguanylic acid (c-di-GMP), which is not produced in archaea or eukaryotes, was first discovered as an allosteric effector of cellulose synthase in *Gluconacetobacter xylinus* and is now recognized as one of the most important and widespread second messengers in bacteria. c-di-GMP is synthesized from two molecules of GTP by diguanylate cyclases (DGCs), which are characterized by active site GGDEF motifs (A-site) (Paul et al., 2004, Chan et al., 2004). The majority of active DGCs also carry a so-called inhibitory or I-site motif, RxxD, which is involved in feedback inhibition (Christen et al., 2006, Schirmer and Jenal, 2009). Specific phosphodiesterases (PDEs), which harbor EAL or HD-GYP domains, degrade the cyclic dinucleotide (Schmidt et al., 2005, Christen et al., 2005, Ryan et al., 2006). The enzymatically active domains involved in c-di-GMP turnover are often associated with diverse sensory domains, thus enabling cells to adjust second messenger levels in response to different environmental stimuli (Hengge, 2009).

The binding of c-di-GMP to effector proteins impacts diverse processes such as adhesion, virulence, motility, and biofilm formation in unicellular, flagellated bacteria (Römling et al., 2013). The known c-di-GMP-binding motifs of these proteins are limited but include degenerate GGDEF domain proteins carrying I-site motifs (Duerig et al., 2009, Lee et al., 2007b, Petters et al., 2012), inactive EAL domain receptors (Navarro et al., 2009, Qi et al., 2011, Newell et al., 2009), and PilZ domain-containing proteins (Amikam and Galperin, 2006). Transcription factors that sense c-di-GMP lack these common c-di-GMP-binding motifs and thus must be identified experimentally. The sparse list of known c-di-GMP-responsive transcriptional regulators includes the TetR-like activator LtmA from *Mycobacterium smegmatis* (Li and He, 2012), the CRP-FNR-like transcription factor Clp from *Xanthomonas* (Chin et al., 2010, Leduc and Roberts, 2009), Bcam1349 from *Burkholderia* (Fazli et al., 2011), the NtrC-type protein FleQ from *Pseudomonas aeruginosa* (Baraquet and Harwood, 2013), and VpsR from *Vibrio cholerae* (Srivastava et al., 2011). The only c-di-GMP-responsive transcription factor for which structural information is available and hence c-di-GMP binding is understood is VpsT, which is a member of the well-studied FixJ-LuxR-CsgD family of response regulators. The VpsT structure revealed a characteristic response regulator fold and a W(F/L/M)(T/S)R c-di-GMP-binding motif (Krasteva et al., 2010). Notably, in all known structures of c-di-GMP-binding effector proteins or enzymes, the c-di-GMP is bound either as a monomer or intercalated dimer. Biophysical studies suggest the possibility of higher order oligomeric forms of c-di-GMP, but they have yet to be observed in any biological context (Gentner et al., 2012).

**Figure 1:**
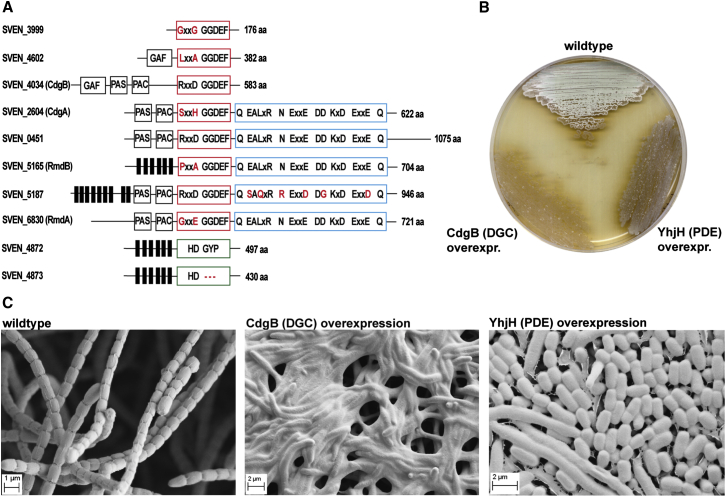
c-di-GMP Levels Affect *S. venezuelae* Development (A) Domain organization of predicted active c-di-GMP-metabolizing proteins in *S. venezuelae*. GGDEF domains are shown as red boxes. Amino acids different from the conserved product inhibition site (RxxD) are shown in red. Noncanonical residues of the EAL domains (blue box) and HD-GYP domains (green box) are highlighted in red. Predicted transmembrane helices are shown as black bars, and N-terminal GAF, PAS, and PAC signaling domains are boxed in black. (B) Overexpression of CdgB (a DGC from *S. coelicolor*) or YhjH (a PDE from *E. coli*) from the *ermEp*^∗^ promoter in *S. venezuelae* results in loss of aerial mycelium formation. (C) SEMs reveal that while CdgB overexpression blocks development (giving rise to a classic “bald” phenotype; middle), YhjH overexpression (right) induces precocious hypersporulation without formation of aerial hyphae. Spore-bearing aerial hyphae of the WT are shown for comparison (left). Cells were grown on maltose-yeast extract-malt extract (MYM) agar for four days at 30°C prior to imaging. See also Figure S1.

While the roles played by c-di-GMP in controlling cellular processes in unicellular bacteria are becoming clear, the function(s) of c-di-GMP in multicellular, nonmotile bacteria such as *Streptomyces* are unknown. The complex *Streptomyces* life cycle involves two distinct filamentous cell forms: the growing or vegetative hyphae and the reproductive or aerial hyphae, which differentiate into exospores for dispersion through a massive synchronous septation event (Flärdh and Buttner, 2009). In the model species *Streptomyces venezuelae,* there are three GGDEF proteins, two proteins with HD-GYP domains, and five proteins containing both a GGDEF and an EAL domain (Figure 1A). Altered expression of the GGDEF proteins, CdgA and CdgB, and deletions of the EAL proteins, RmdA and RmdB, have a significant impact on *Streptomyces* growth progression, suggesting that c-di-GMP plays a role in controlling developmental processes in multicellular bacteria (den Hengst et al., 2010, Tran et al., 2011, Hull et al., 2012). Interestingly, *cdgA* and *cdgB* have recently been identified as direct regulatory targets of the developmental master regulator BldD (den Hengst et al., 2010, Tran et al., 2011). Mutations in the *bld* loci block the formation of aerial hyphae, resulting in a “bald” phenotype, and also affect the production of antibiotics (McCormick and Flärdh, 2012).

BldD sits at the top of the regulatory cascade controlling development, serving to repress expression of sporulation genes during vegetative growth (den Hengst et al., 2010). In *Streptomyces coelicolor*, BldD controls the expression of at least 167 genes, including 42 genes (∼25% of the regulon) that encode regulatory proteins (Elliot et al., 2001, den Hengst et al., 2010). Among these BldD targets are many genes known to play critical roles in *Streptomyces* development, including other *bld* regulators (e.g., *bldA*, *bldC*, *bldH*/*adpA*, *bldM*, and *bldN*), several *whi* (white) regulators required for the differentiation of aerial hyphae into spores (e.g., *whiG* and *whiB*), and genes encoding critical components of the cell division and chromosome segregation machineries such as FtsZ, SsgA, SsgB, and the DNA translocase SffA (den Hengst et al., 2010, McCormick, 2009). How BldD activity is regulated, however, has been unknown.

Here we show that BldD is a c-di-GMP-binding effector protein, thus revealing a link between c-di-GMP signaling and the development of multicellular bacteria. Specifically, structural and biochemical analyses show that the second messenger c-di-GMP activates BldD DNA binding by driving a unique form of dimerization that is mediated by a tetrameric form of c-di-GMP. The c-di-GMP tetramer performs its oligomerization function by adjoining two BldD C-terminal domain (CTD) protomers, the polypeptide chains of which are separated by ∼10 Å. BldD recognizes the c-di-GMP tetramer using a bipartite RXD-X_8_-RXXD c-di-GMP interaction signature sequence from each subunit. Thus, tetrameric c-di-GMP acts as a small-molecule dimerizing agent that controls the DNA-binding activity of BldD, leading to repression of the BldD regulon of sporulation genes during vegetative growth, thereby controlling the hypha-to-spore transition in multicellular bacteria.

## Results

### c-di-GMP Controls Developmental Program Progression in *Streptomyces venezuelae*

**Figure S1:**
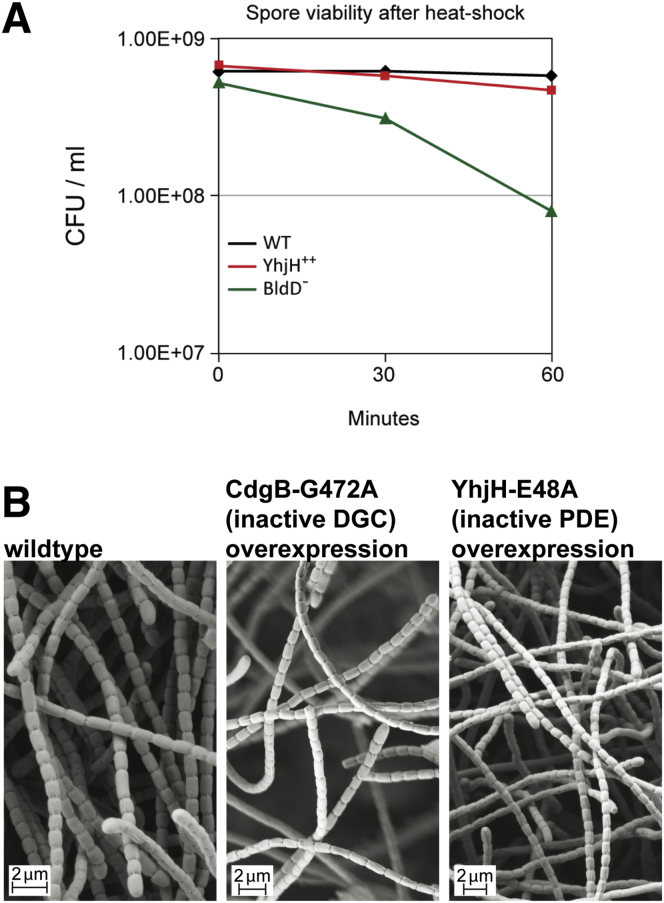
Robustness of Spores Made by the YhjH-Overexpression Strain and the *bldD* Mutant and Overexpressing Catalytically Inactive Versions of CdgB and YhjH Has No Effect on Development, Related to Figure 1 (A) Spore heat resistance was determined as described previously (Molle et al., 2000). The *bldD* mutant spore defect is mild; for comparison, under the same conditions (50°C) *whiD* mutant spore viability drops by a factor of 10^8^ in 30 min (Molle et al., 2000). (B) Scanning electron micrographs showing that overexpression of catalytically inactive versions of YhjH or CdgB has no effect on *S. venezuelae* development. A tag-less variant of *cdgB* carrying a G472A mutation in the GGDEF motif was PCR-amplified from pIJ10361 (Tran et al., 2011). CdgB-AGDEF and the inactive variant of YhjH with the E48A mutation in the EAL-motif (Pesavento et al., 2008) were used for overexpression from the *ermEp*^∗^ promoter. Cells were grown on MYM for 4 days at 30°C.

To gain insight into the cellular processes controlled by c-di-GMP in streptomycetes, we overexpressed either the active DGC CdgB from *S. coelicolor* (Tran et al., 2011) or the active PDE YhjH from *E. coli* (Pesavento et al., 2008). Strikingly, overexpression of both CdgB and YhjH blocked the generation of aerial mycelium by *S. venezuelae* (Figure 1B). However, scanning electron micrographs (SEMs) revealed that, whereas overexpression of CdgB blocked development, resulting in a classical bald phenotype, overexpression of the PDE YhjH in fact promoted sporulation, but the colonies appeared bald to the naked eye because aerial mycelium formation had been bypassed (Figure 1C). As judged by heat resistance, the spores made by the YhjH overexpression strain were as robust as those of the wild-type (WT) (Figure S1A available online). Moreover, overexpression of catalytically inactive versions of YhjH or CdgB had no effect on *S. venezuelae* development (Figure S1B). These data suggest that intracellular levels of c-di-GMP influence the timing of development. In particular, they suggest that increased c-di-GMP levels delay differentiation, arresting the colonies in the vegetative growth stage, whereas decreased levels of the second messenger accelerate development, favoring sporulation.

### BldD Is a c-di-GMP Effector Protein

*S. venezuelae* has no PilZ domain-containing proteins, and no putative c-di-GMP-binding effector proteins have so far been identified in the *Streptomyces* genus. Thus, to address the mechanism by which *S. venezuelae* senses c-di-GMP to control sporulation, we sought to identify c-di-GMP effector proteins involved in development. To selectively enrich putative c-di-GMP-binding proteins from *S. venezuelae* cell extracts, we performed an affinity pull-down assay using a c-di-GMP capture compound (Nesper et al., 2012). Captured proteins were identified by tryptic mass spectrometry fingerprinting.

**Figure 2:**
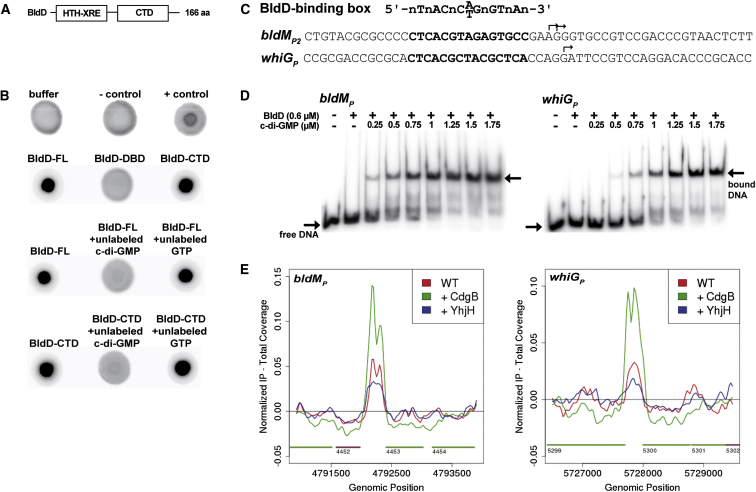
The BldD CTD Binds c-di-GMP and Activates DNA Binding (A) Schematic showing the domain organization of the BldD protein. BldD contains an N-terminal DBD connected by a flexible linker to a CTD. (B) Results of DRaCALAs assays carried out using purified FL His_6_-BldD, His_6_-BldD-DBD or His_6_-BldD-CTD, and ^32^P-labeled c-di-GMP. The TetR-like regulator SVEN_1547 and the active DGC PleD^∗^ from *C. crescentus* served as negative (−) and positive (+) controls, respectively (top row). In competition DRaCALAs, excess cold c-di-GMP or GTP was added to the binding reaction containing ^32^P-labeled c-di-GMP and His_6_-FL BldD or His_6_-BldD-CTD. c-di-GMP binding is indicated by dark spots centered on the nitrocellulose. (C) Top: the DNA consensus motif bound by BldD (den Hengst et al., 2010). Below are the sequences from the *S. venezuelae bldM* and *whiG* promoter regions (with BldD-binding boxes in bold). The transcriptional start sites are indicated by bent arrows. (D) EMSA analyses of BldD binding to the *bldM* and *whiG* promoters ± c-di-GMP. Free DNA and protein-DNA complexes (bound DNA) are indicated with arrows. (E) In vivo BldD ChIP-seq data for *bldM* and *whiG*. Color coding of the ChIP samples is as follows: WT *S. venezuelae* (red), *S. venezuelae* overexpressing the *S. coelicolor* DGC CdgB (green), and *S. venezuelae* overexpressing the *E. coli* PDE YhjH (blue). Plots span ∼3 kb of DNA sequence. Genes running left to right are shown in green, and genes running right to left are shown in red.

**Figure S2:**
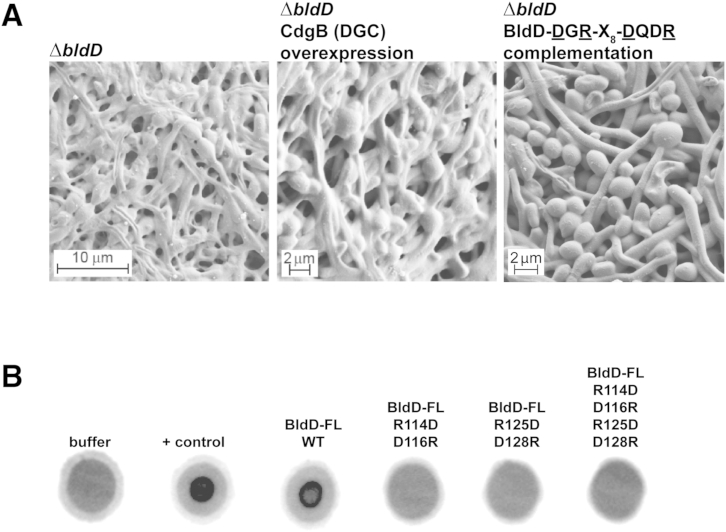
CdgB Overexpression Has No Phenotypic Effect on a *bldD* Mutant, c-di-GMP Binding Is Required for BldD Activity In Vivo, and Motifs 1 and 2 Are Both Required for c-di-GMP Binding In Vitro, Related to Figures 3 and 5 (A) Scanning electron micrographs showing that overexpression of CdgB has no effect on the phenotype of the *bldD* mutant and that an allele of *bldD* encoding a protein defective in c-di-GMP binding (with the DGR-X_8_-DQDR mutation) cannot complement a *bldD* mutant. CdgB was overexpressed from the *ermEp^∗^* promoter and the mutagenized *bldD* gene was expressed from its native promoter. Strains were grown for 40 hr at 30°C. (B) BldD CTD Motifs 1 and 2 are both required for c-di-GMP binding. The double mutants DGR-X_8_-RQDD (Motif 1 mutated) and RGD-X_8_-DQDR (Motif 2 mutated), in which R114 and D116 or R125 and D128 were changed to D and R, respectively, as well as the quadruple mutant, DGR-X_8_-DQDR, (R114, D116, R125 and D128 of Motifs 1 and 2 were changed to D, R, D and R, respectively) of BldD Full-Length (FL) were generated using a four-primer/two-step PCR protocol (Germer et al., 2001) and tested in DRaCALAs (Roelofs et al., 2011) for c-di-GMP binding.

Remarkably, the developmental master regulator BldD was repeatedly recovered in our c-di-GMP-based capture compound experiments. BldD is an 18 kDa DNA-binding protein (Elliot and Leskiw, 1999) consisting of two distinct domains connected by a flexible linker (Figure 2A). The N-terminal domain is the DNA-binding domain (DBD) and has a xenobiotic response element (XRE) helix-turn-helix (HTH) DNA-binding motif (Kim et al., 2006). The BldD CTD harbors a largely helical fold with no known function (Kim et al., 2014). To probe the interaction between BldD and c-di-GMP further and to identify the c-di-GMP-binding domain, we used differential radial capillary action of ligand assays (DRaCALA) (Roelofs et al., 2011). DRaCALA allows the visualization of protein-bound radiolabeled ligand as a concentrated spot after the application of the protein-ligand mixture onto nitrocellulose. Using this assay, we confirmed that full-length (FL) BldD (expressed as an N-terminally His_6_-tagged protein) from both *S. venezuelae* (Figures 2B and S2B) and *S. coelicolor* (data not shown) bind ^32^P-labeled c-di-GMP. Importantly, the DRaCALA assays demonstrated that the previously uncharacterized CTD of BldD functions as the c-di-GMP-binding domain (Figure 2B). Further, excess unlabeled c-di-GMP, but not GTP, competed with the labeled c-di-GMP for binding to FL BldD and to BldD-CTD. Thus, these data reveal that the CTD of the key developmental regulator BldD is a c-di-GMP-binding domain.

### Cyclic di-GMP Enhances Binding of BldD to Its Target Promoters In Vitro and In Vivo

Using global chromatin immunoprecipitation-microarray analysis (ChIP-chip), we previously identified the complete BldD regulon in *S. coelicolor*, showing that it encompasses ∼167 transcription units (den Hengst et al., 2010). Through MEME-based sequence analysis of all the promoter regions directly targeted by BldD, we defined a 13 bp pseudo-palindromic sequence, 5′-TNAC(N)_5_GTNA-3′, designated the BldD box, which functions as a specific binding sequence for BldD (den Hengst et al., 2010). Sequence analysis showed that the BldD box was conserved between *S. coelicolor* and *S. venezuelae* for most key BldD target promoters (den Hengst et al., 2010). Further, BldD from *S. coelicolor* and *S. venezuelae* contain an identical DBD and differ by only two residues in the DBD-CTD linker and five residues in the CTD, suggesting that BldD function is broadly conserved between the two species.

Having shown that BldD binds c-di-GMP, we tested the effect of c-di-GMP on BldD DNA binding. Radiolabeled *S. venezuelae* DNA fragments encompassing the promoter regions of two well-characterized BldD target genes, *bldM* and *whiG*, including the bioinformatically identified BldD box (Figure 2C), were used as target DNAs in electrophoretic mobility shift assays (EMSAs) (Figure 2D). The fixed concentration of BldD used in these assays (0.6 μM) was insufficient to elicit a DNA mobility shift. However, the addition of increasing concentrations of c-di-GMP (0.25–1.75 μM) strongly induced BldD binding to both the tested promoter regions (Figure 2D). To confirm and extend these results into cells, we manipulated the levels of c-di-GMP in *S. venezuelae* and monitored the effect on BldD binding to the *bldM* and *whiG* promoters in vivo using ChIP-sequencing (ChIP-seq). The degree of BldD binding was assayed at a single time point in WT *S. venezuelae* and the WT overexpressing either the DGC CdgB or the PDE YhjH (the strains whose phenotypes are described above). Consistent with the in vitro EMSA data, overexpression of the DGC enhanced ChIP-seq peak height at the BldD target promoters relative to the WT control (Figure 2E). Conversely, overexpression of the PDE lowered ChIP-seq peak heights at BldD target promoters relative to the WT (Figure 2E). These data demonstrate that c-di-GMP enhances the binding of BldD to the BldD box, stimulating BldD-mediated repression of its target regulon. Thus, it is not BldD, but a BldD-(c-di-GMP) complex, that serves to turn off sporulation genes during vegetative growth.

### *S. venezuelae bldD* Mutants Show an Accelerated Sporulation Phenotype that Bypasses Aerial Mycelium Formation

**Figure 3:**
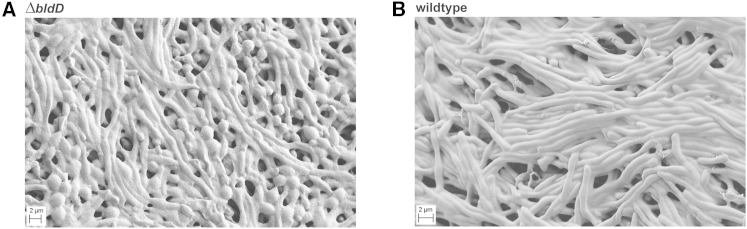
BldD-Deficient Cells Show an Enhanced Sporulation Phenotype (A and B) SEMs showing that a constructed *S. venezuelae ΔbldD* mutant (A) exhibits accelerated sporulation compared to the WT (B). At 36 hr the WT strain consists of pure vegetative mycelium. Strains were grown on MYM agar for 36 hr at 30°C prior to imaging. See also Figure S2A.

The opposing effects of the overexpression of the DGC CdgB and the PDE YhjH suggested that high levels of c-di-GMP retard sporulation and low levels of c-di-GMP accelerate sporulation. Because the BldD-(c-di-GMP) complex serves to keep sporulation genes shut off during vegetative growth, loss of BldD should have a similar effect on *Streptomyces* development as depletion of c-di-GMP levels. To test this hypothesis, we deleted *bldD* from the *S. venezuelae* chromosome. Strikingly, the *bldD* null mutant formed small colonies lacking aerial hyphae, but—when examined by SEM—even young colonies of the *bldD* mutant were found to contain spore chains embedded in an excess of extracellular matrix (Figure 3A). Heat resistance tests showed the *bldD* mutant spores were mildly defective (Figure S1A). By contrast, equivalent young colonies of the WT that were grown and imaged in parallel had not yet developed aerial hyphae or spores (Figure 3B). Thus, loss of BldD mimics the effects of overexpressing the PDE YhjH (compare Figures 1C and 3A). In addition, overexpression of CdgB had no effect on the phenotype of the *bldD* mutant (Figure S2A), further supporting the idea that c-di-GMP signals through BldD to control the hypha-to-spore transition.

### Crystal Structures of BldD CTD-(c-di-GMP) Complexes: Small-Molecule-Mediated Protein Dimerization

**Figure 4:**
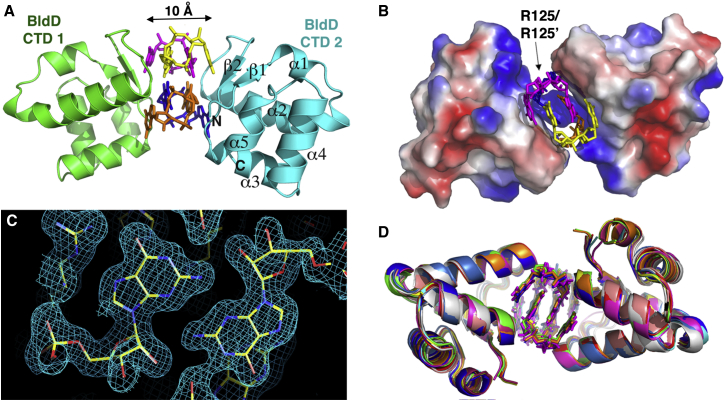
Structures of BldD CTD-(c-di-GMP) Complexes Reveal a Unique c-di-GMP Oligomer and c-di-GMP-Mediated Dimerization Mechanism (A) Ribbon diagram of the *S. venezuelae* BldD CTD-(c-di-GMP) complex. Secondary structural elements of CTD2 are labeled. The c-di-GMP tetramer is shown as sticks with each c-di-GMP molecule colored differently. Ribbon diagrams were made using PyMol (Delano, 2002). (B) Electrostatic surface representation of the BldD CTD-(c-di-GMP) complex. Blue and red represent electropositive and electronegative surfaces, respectively. The four c-di-GMP molecules are colored as in (A). (C) Composite 2F_o_-F_c_ omit map of the 1.75 Å BldD CTD-c-di-GMP complex contoured at 1σ around the central nucleotides of the c-di-GMP tetramer. (D) Superposition of all 12 Bld CTD-(c-di-GMP) complexes (rainbow colored) determined in this study. See also Figures S3 and S4 and Tables S1 and S2.

To elucidate the molecular basis for c-di-GMP recognition and binding by the BldD CTD and to gain insight into how this interaction may elicit developmental signaling, we determined structures of the *S. venezuelae* BldD CTD (residues 80–166) and *S. coelicolor* BldD CTD (residues 80–167) in complex with c-di-GMP. Three *S. venezuelae* BldD C-domain-(c-di-GMP) structures and one *S. coelicolor* BldD C-domain-(c-di-GMP) structure were determined to resolutions of 1.95 Å, 2.33 Å, 1.75 Å, and 2.25 Å, respectively (Figure 4) (see Extended Experimental Procedures and Tables S1 and S2).

**Figure S3:**
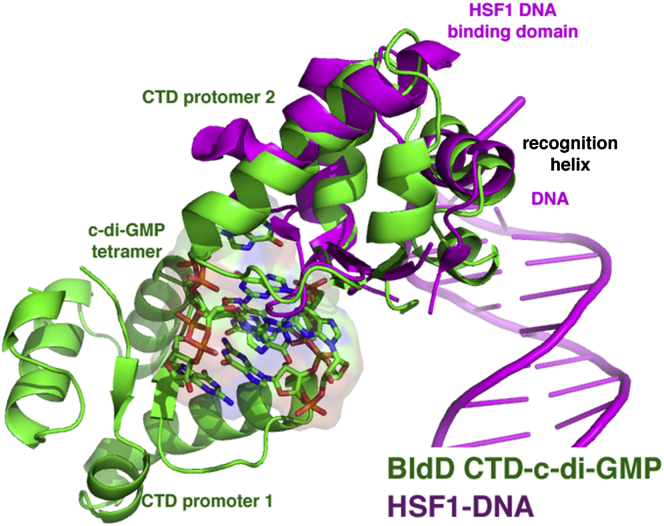
Superposition of the BldD CTD and the Human HSF1 DNA-Binding Winged HTH Motif, Related to Figure 4 The resulting root mean square deviation of 52 corresponding Cα atoms is 2.4 Å. The HSF1-DNA structure is magenta and the BldD CTD is green and the bound c-di-GMP tetramer is shown as sticks. Note that the nucleotide binding motifs are entirely different. The PDB code for the human HSF1 DNA-binding winged HTH motif is 1FYL.

The BldD CTD structures are composed of two (β-α-α) repeats followed by a short C-terminal helix and are similar to the apo form studied by NMR (Kim et al., 2014) (Figure 4). Database searches revealed that the BldD CTD harbors a new fold, but reduced stringency revealed that it shows limited structural similarity with winged HTH proteins, in particular, the winged HTH domain of eukaryotic heat shock factor 1 (HSF1) (Littlefield and Nelson, 1999). The BldD CTD and HSF1 bind c-di-GMP and DNA, respectively, but the motifs they employ to interact with their nucleotide ligands are completely different (Figure S3). The BldD CTD uses a previously unseen mode of c-di-GMP binding in which two noninteracting CTDs are glued together by a c-di-GMP tetramer composed of two interlocked c-di-GMP dimers (Figure 4A). Thus, in the BldD CTD-(c-di-GMP) complex, c-di-GMP functions as a macromolecular dimerizer. Indeed, the closest approach of any Cα atoms of the two tethered CTDs is ∼10 Å.

**Figure 5:**
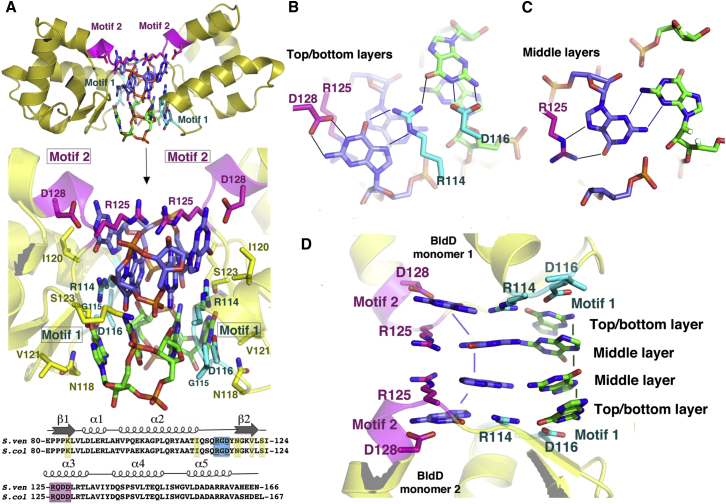
The BldD CTD Contains a c-di-GMP-Binding Signature Composed of Two Contiguous Motifs (A) Structure of the BldD dimer-(c-di-GMP) complex. Top: the location of the two c-di-GMP-binding motifs (motif 1 and motif 2) of the bridged CTD promoters. Middle: a close-up of selected residues of the BldD CTD that interact with the c-di-GMP nucleotides. Bottom: alignment of the sequences of the *S. venezuelae* and *S. coelicolor* BldD CTDs. Motif 1 is colored cyan and motif 2 is magenta. Other residues that contact c-di-GMP are colored yellow. (B) Close-up of the top and bottom layer CTD-(c-di-GMP) contacts, including the Arg114 side chain-guanine interactions, which anchor the two intercalated dimers together. (C) Close-up of the central or middle layers of the two intercalated c-di-GMP dimers. Shown are the hydrogen bonds from R125 and one guanine and those between the N2 and N3 atoms of two guanines from different c-di-GMP dimers that tether the intercalated dimers in these layers. (D) Side view of the c-di-GMP layers, highlighting the multiple base-base, side chain-base, and side chain-side chain stacking interactions that stabilize the c-di-GMP tetramer structure. Base stacking is depicted by appropriately colored solid lines. See also Figures S2B, S5, and S7 and Tables S1 and S2.

The BldD CTD interacts with c-di-GMP using two contiguous surface motifs, herein called motif 1 and motif 2, which are located between the two β-α-α repeats. Motif 1 is composed of residues 114–116 (RGD) and motif 2 is composed of residues 125–128 (RQDD) (Figure 5A). These motifs are located on a solvent exposed region at one end of each CTD protomer and not within a pocket or cavity (Figures 4A and 5A). The combined motifs from two CTD subunits provide ideal shape and electrostatic complementarity for encasing the unusual cage-like c-di-GMP tetramer (Figures 4A and 4B). Strikingly, although the BldD CTD is overall acidic (pI∼5.0), the c-di-GMP-binding surface between two BldD protomers is electropositive (Figure 4B). In addition to shape and charge complementary, contacts from the arginine and aspartic acid residues within motifs 1 and 2 provide specificity for recognition of guanine cyclic nucleotides; the multiple interactions effectively exclude binding to adenine cyclic nucleotides. Specifically, motif 2 from each CTD protomer combine to mediate contacts to one intercalated c-di-GMP dimer, Asp116 of motif 1 from each protomer combine to mediate contacts to the other c-di-GMP dimer, while Arg114 sits centrally and anchors both dimers (Figures 5A and 5B). The guanine bases wedged between the two motif 2 regions are specified by contacts from residues Arg125 and Asp128 (Figures 5A and 5B). Residue Asp128 makes two hydrogen bonds to the N1 and the exocyclic N2 atoms of the guanine bases on each end (top and bottom layers) of the intercalated dimer, while Arg125 flanks the guanines in the center (middle layers) of the dimer and makes hydrogen bonds to the guanine O6 and N7 atoms (Figures 5B–5D). The O6 moiety is also specified by the backbone nitrogen of Arg125. Notably, the stacking interactions between the Arg125 side chains are the only direct contacts between the two CTD protomers but are clearly not sufficient to promote BldD dimerization.

Arg114 is the only CTD residue that makes contacts to both intercalated c-di-GMP dimers. Arg114 hydrogen bonds to the guanines contacted by Asp128, as well as the O6 atoms of the adjacent guanines of the other c-di-GMP dimer (Figures 5A and 5B). Thus, Arg114 plays a key role in the recognition and stabilization of this unique c-di-GMP tetramer. The c-di-GMP dimer bound between the motif 1 regions has fewer contacts and is more exposed (Figure 5A). In addition to contacts from Arg114, Asp116 of motif 1 hydrogen bonds to the guanine N1 and N2 atoms in a manner analogous to the contacts from Asp128 of motif 2 (Figure 5). Notably, the specific hydrogen bonds from motif 1 and 2 residues to guanine exocyclic O6 and N2 atoms dictate that BldD binds to c-di-GMP but not to c-di-AMP, which is missing an exocyclic atom at the 2 position and harbors a hydrogen bond donor rather than an acceptor at the exocyclic 6 position. BldD motifs 1 (RXD) and 2 (RXXD), although similar in sequence to the inhibitory I-site (RXXD), which is involved in product inhibition feedback control of DGC activity (Christen et al., 2006), are structurally different. Further, mutagenesis of either BldD motif 1 or motif 2 abolishes c-di-GMP binding in DRaCALA assays (Figure S2B), confirming that, unlike I-site c-di-GMP binding, both motifs 1 and 2 in BldD are required to construct the complete binding site for the tetrameric c-di-GMP complex. This dual signature sequence is unlike any previously characterized c-di-GMP-binding motif. Moreover, specific binding of the c-di-GMP tetramer requires encasement by two such bipartite motifs from precisely oriented BldD protomers. While the arginine and aspartic acid residues in BldD motifs 1 and 2 dictate the c-di-GMP-binding arrangement and read the guanine bases, contacts to the c-di-GMP phosphate groups are provided by Lys84 from β1 and Arg130 from α3. Further, Ile110 makes van der Waals interactions and residues Asn118 and Ser123 hydrogen bond with the guanine bases on the top and bottom layers of the c-di-GMP tetramer (Figure 5A). The combination of the optimally positioned bipartite signature sequences from two BldD protomers exquisitely templates binding of the specific and unusual c-di-GMP tetramer structure.

### The BldD-(c-di-GMP) Structure Reveals a Tetrameric Form of the c-di-GMP Second Messenger

**Figure S4:**
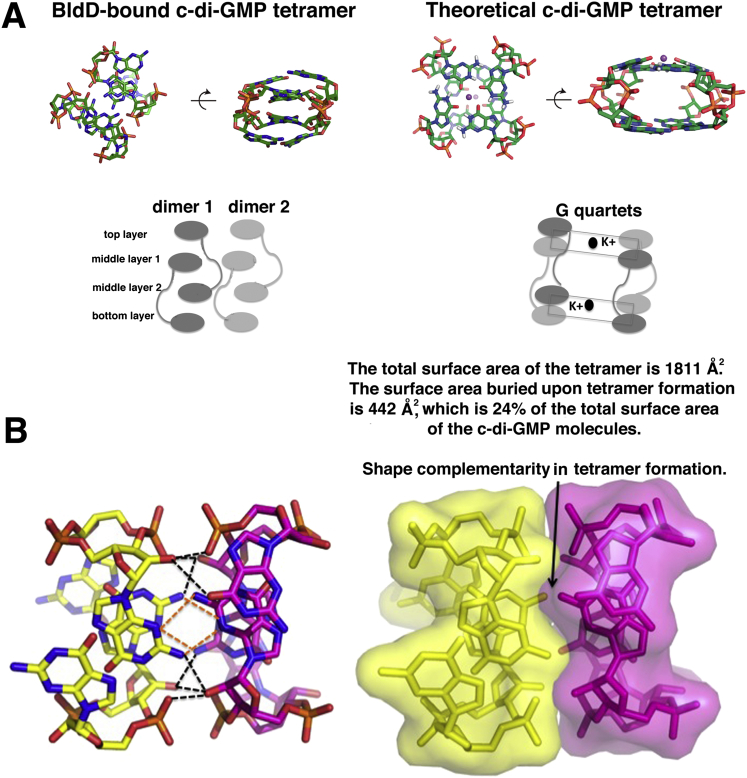
BldD Binds a Tetrameric Form of c-di-GMP, Related to Figure 4 (A) The BldD bound c-di-GMP forms a novel tetrameric structure composed of two intercalated c-di-GMP dimers. This figure compares the BldD bound c-di-GMP tetramer (left) with a proposed c-di-GMP tetramer that forms at high concentration and is favored by the presence of K^+^ ions (right). The theoretical quartet-like structure was proposed from CD and NMR experiments. Below shows a schematic representation of the two, which underscores key differences including the alternating base stacking that is present in the BldD bound tetramer but not the theoretical tetramer and the presence of G-quartet interactions in the theoretical tetramer favored by the K^+^ ions and not present in the BldD bound tetramer. (B) Close up of the BldD bound c-di-GMP tetramer that is formed by the tight interaction between two intercalated c-di-GMP dimers (one c-di-GMP intercalated dimer is colored yellow and the other magenta). Orange dots represent specific hydrogen bonds between bases while black dots are hydrogen bonds between bases and/or ribose and phosphate groups. The left panel shows the 12 hydrogen bonds between the two intercalated dimers within the c-di-GMP tetramer, and the right panel highlights the ideal shape complementarity at this interface.

c-di-GMP is monomeric in solution at physiological concentrations (Gentner et al., 2012). However, intercalated c-di-GMP dimers have been observed in crystal structures of the nucleotide alone and in complexes with effector proteins. Higher order c-di-GMP structures such as tetramers and octamers have thus far only been inferred from NMR and spectroscopic studies and require very high c-di-GMP concentrations (up to 30 mM) and monovalent cations (Zhang et al., 2006). These higher order structures are characterized by G-quartet interactions with a centrally bound potassium ion coordinated by four guanines. There are minimal base contacts and no base stacking interactions in these structures (Figure S4A) (Zhang et al., 2006, Gentner et al., 2012). By sharp contrast, the BldD-bound tetrameric c-di-GMP is a tightly packed structure that is not secured by ions. Rather, the c-di-GMP molecules are closely spaced and optimally positioned for interbase pairing, leading to the formation of a multistranded, base-stacked structure with top, middle, and bottom layers (Figures 5D and S4A). There are 12 hydrogen bonds between the two intercalated dimers within the c-di-GMP tetramer, including contacts between the N3 atoms and exocyclic NH_2_ amides of an adjacent base (Figures 5C and S4B). Such contacts could not be formed with c-di-AMP due to its lack of an exocyclic NH_2_ atom. Therefore, in addition to contacts from motifs 1 and 2, guanine-guanine base hydrogen bonds serve to specify c-di-GMP tetramer binding to BldD. Notably, formation of the c-di-GMP tetramer buries 24% of the total surface area (buried surface area [BSA]) of the c-di-GMP molecules (Figure S4B). By comparison, in most protein oligomers the BSA between protomers is ∼15% (Wang et al., 2009). Finally, the interface between the intercalated c-di-GMP dimers that forms the tetramer is remarkably complementary in shape (Figure S4B). Thus, the combination of multiple contacts between the c-di-GMP moieties along with its extensive BSA and molecular shape complementarity lead to the creation of a compact and highly specific c-di-GMP tetramer. However, BldD is necessary to stabilize this tetramer and template its formation.

### c-di-GMP Induces Dimerization of the BldD CTD in Solution

**Figure 6:**
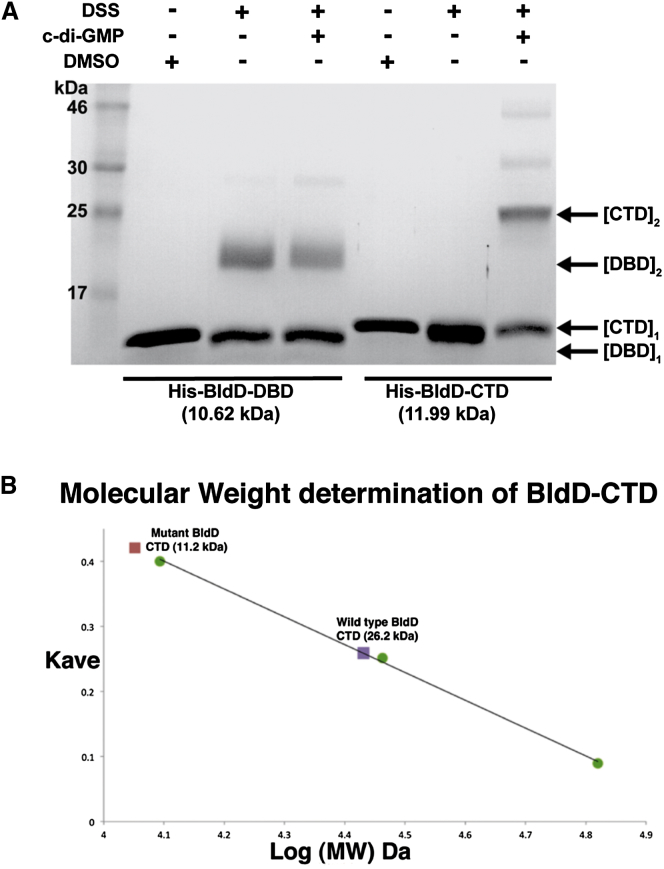
c-di-GMP Is Required for BldD CTD Dimerization in Solution (A) DSS chemical crosslinking. We crosslinked 10 μM His_6_-BldD-DBD (10.7 kDa) or His_6_-BldD-CTD (12.0 kDa) by using 1 mM DSS in the presence or absence of 20 μM c-di-GMP as indicated. In control reactions, the DSS solvent dimethylsulfoxide (DMSO) alone was added to the proteins. Samples were analyzed by SDS-PAGE. Monomers and dimers of each BldD domain are marked by arrows. (B) SEC analysis of the WT BldD CTD and BldD CTD DGR-X_8_-DQDR mutant in the presence of 3 μM c-di-GMP. The WT CTD + c-di-GMP is dimeric, while the quadruple mutant is monomeric. The standard curve was determined using cytochrome C (12 kDa), carbonic anhydrase (29 kDa), and albumin (66 kDa) (green circles).

The BldD CTD-(c-di-GMP) crystal structures reveal that c-di-GMP acts as a “dimerizer” to link two CTD protomers. To examine the effect of c-di-GMP on the oligomeric state of the BldD CTD in solution, we carried out chemical crosslinking and size exclusion chromatography (SEC) studies. Chemical crosslinking experiments were performed using disuccinimidyl suberate (DSS), which contains amine-reactive N-hydroxysuccinimide esters at both ends of an 11 Å spacer arm. This reagent should therefore be able to crosslink even the distantly anchored CTD protomers observed in our CTD-(c-di-GMP) structures (Figure 4A). In the absence of DSS, the BldD DBD and CTD migrate on SDS-PAGE gels as single bands with the expected monomeric molecular weights of 11 and 12 kDa, respectively (Figure 6A). Upon incubation with DSS, the BldD DBD forms a covalent dimer of ∼21 kDa, consistent with previous biochemical and structural analyses of this domain (Kim et al., 2006, Lee et al., 2007a), and oligomerization is unaffected by the addition of c-di-GMP (Figure 6A). By contrast, the BldD CTD remains monomeric after DSS addition. However, in the presence of c-di-GMP, addition of DSS results in the clear formation of covalent CTD dimers (Figure 6A).

To examine c-di-GMP-induced CTD oligomerization further, we performed SEC analyses. As part of this study, we mutagenized the RGD (motif 1)-X_8_-RQDD (motif 2) c-di-GMP-binding signature of the BldD CTD. To retain the charge of these surface residues, we made the charge-swapped DGR-X_8_-DQDR CTD mutant, changing the key Arg residues within the two motifs to Asp, and vice versa. DRaCALA assays showed that this mutant CTD was unable to bind c-di-GMP (Figure S2B). SEC experiments in the presence of 3 μM c-di-GMP showed that the WT CTD forms a dimer, while the mutant CTD is monomeric (Figure 6B). Hence, the crosslinking and SEC data support our structural finding that c-di-GMP is required for BldD CTD dimerization. Further, we constructed a *bldD* mutant allele encoding a protein solely defective in c-di-GMP binding (carrying the DGR-X_8_-DQDR mutation) and found that it had no ability to complement a *bldD* mutant (Figure S2A), confirming that the major in vivo functions of BldD are indeed mediated by its binding to c-di-GMP.

### Affinity, Stoichiometry, and Specificity of c-di-GMP for BldD CTD

**Figure S5:**
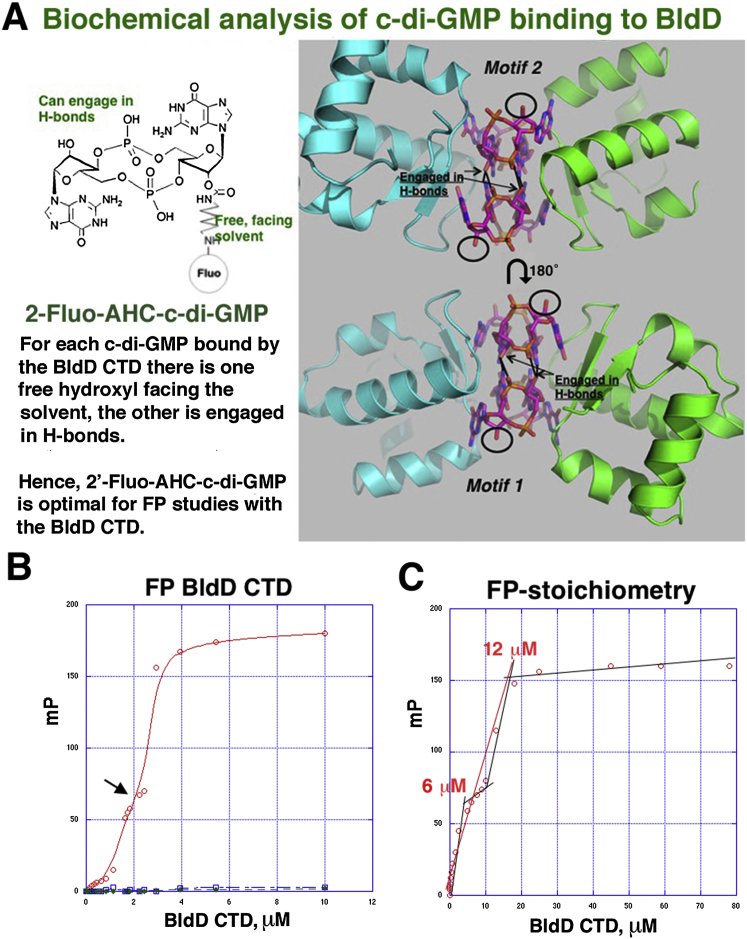
Determination of BldD CTD Affinity, Stoichiometry, and Specificity for c-di-GMP by Fluorescence Polarization, Related to Figure 5 (A) Left is the singly fluoresceinated probe, 2′-Fluo-AHC-c-di-GMP, used in FP binding experiments. Right, the BldD-c-di-GMP structure shows that one ribose of each c-di-GMP must be unmodified to permit hydrogen bonding (arrowed), while the other is free (ringed), making 2′-Fluo-AHC-c-di-GMP an optimal probe for binding studies. (B) Binding isotherm of WT BldD CTD (red circles), and the Motif1/Motif2 (DGR-X_8_-DQDR) CTD mutant (blue squares) to 2′Fluo-AHC-c-di-GMP. The green squares show the binding isotherm of WT BldD CTD to 2′-Fluo-AHC-c-di-AMP. The results reveal that WT BldD binds c-di-GMP with high affinity but shows no binding to c-di-AMP and that the BldD DGR-X_8_-DQDR mutant does not bind c-di-GMP. The K_d_ for the WT BldD CTD binding to c-di-GMP was 2.5 μM. A possible initial binding event (at 1.7 μM) is indicated by a black arrow. (C) Determination of the stoichiometry of the BldD CTD-(c-di-GMP) complex. To determine the binding stoichiometry, the same FP conditions as B were used but with 25 μM c-di-GMP (total concentration) added to the binding reaction, a concentration which is ∼10-fold above the K_d_ and a necessity for proper determination of the binding stoichiometry. The graph of the resulting data shows a linear increase in the observed mPs until saturation of the binding sites, after which the binding curve flattens. The inflection point can be observed at a BldD protomer concentration of 12 μM, which, when divided by the concentration of c-di-GMP (25 μM), indicates a stoichiometry of two CTD protomers per four c-di-GMPs. There is a possible inflection point at 6 μM, which would indicate a binding stoichiometry of two c-di-GMPs/CTD dimer (correlating with the possible initial binding event in B). These two very close binding events are consistent with positive cooperativity.

Our structures reveal a unique interaction between the BldD CTD and a c-di-GMP tetramer. Remarkably, every guanine in this complex is read specifically by either BldD arginines, aspartic acids, and/or other guanine bases, suggesting that BldD binds only c-di-GMP, and not other cyclic nucleotides. To test this hypothesis and further probe the c-di-GMP-binding affinity of BldD, we performed fluorescence polarization (FP) experiments. These studies were performed with BldD CTD that was expressed and purified from Sf9 insect cells to ensure no c-di-GMP was present, as eukaryotes do not produce c-di-GMP (Extended Experimental Procedures). The BldD-(c-di-GMP) structures show that one ribose of each c-di-GMP bound to BldD must be unmodified to permit formation of the BldD-(c-di-GMP) complex (Figure S5A). Hence, for these studies we used the fluoresceinated probe, 2′-Fluo-AHC-c-di-GMP, which harbors the fluorescein dye on only one ribose (Figure S5A). These studies revealed BldD CTD bound to 2′-Fluo-AHC-c-di-GMP, with an apparent K_d_ of 2.5 μM ± 0.6 (Figure S5B). Consistent with our DRaCALA assays (Figure S2B), the DGR-X_8_-DQDR CTD mutant failed to bind 2′-Fluo-AHC-c-di-GMP in FP assays (Figure S5B). WT BldD CTD showed no binding to the identically fluoresceinated c-di-AMP tagged molecule, 2′-Fluo-AHC-c-di-AMP (Figure S5B).

Next, to ascertain the stoichiometry of c-di-GMP binding in solution, we used an FP-based binding assay. The resulting data (Figure S5C) show a linear increase in fluorescence polarization until saturation of the binding sites. A single inflection point can be fitted at a BldD monomer concentration of ∼12 μM, which equates to a stoichiometry of four c-di-GMP molecules per CTD dimer. However, careful inspection of the data reveals another potential inflection point at a BldD monomer concentration of 6 μM, which would be consistent with an initial binding event of two c-di-GMP molecules per CTD dimer. This putative initial binding event is also apparent in the equilibrium-binding isotherm yielding an apparent K_d_ of ∼1.7 μM (Figure S5B). Two-step binding is not inconsistent with the structure, but the nearly identical affinities observed for each binding event suggest positive cooperativity. Overall, these data demonstrate unequivocally that c-di-GMP binds the CTD with a stoichiometry of four c-di-GMP molecules per CTD dimer, concordant with our structures. These studies also demonstrate that the BldD CTD binds specifically and with high affinity to c-di-GMP but not c-di-AMP and that both motifs 1 and 2 are essential for this interaction.

### The Mechanism for (c-di-GMP)-Activated DNA Binding by BldD

**Figure S6:**
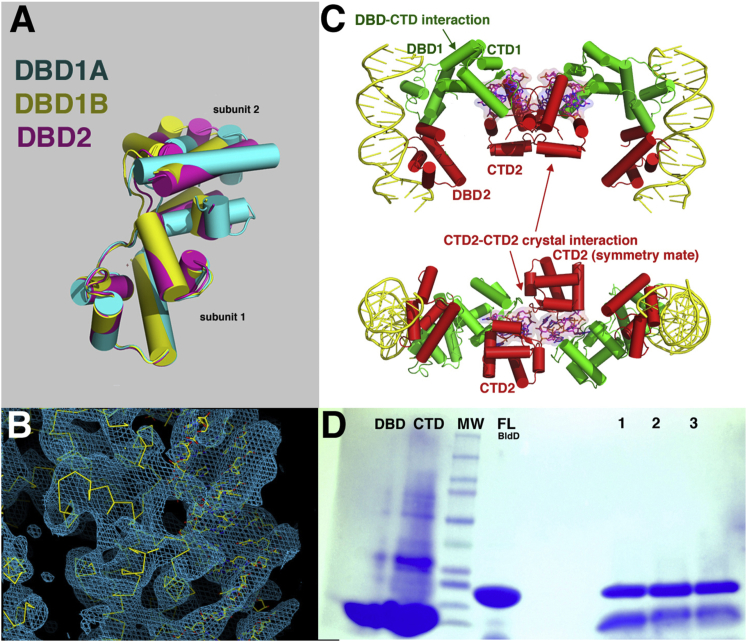
Structural Analyses of the BldD DBD and the Full-Length BldD-(c-di-GMP)-DNA Complex, Related to Figure 7 (A) Overlay of one subunit of the crystallographic dimer of the *S. coelicolor* BldD DBD (yellow) and one subunit of the two crystallographic dimers observed in the *S. venezuelae* DBD structure (magenta and cyan). This overlay shows that the intersubunit interfaces of these dimers are small and likely not physiologically relevant (∼300 Å^2^ BSA buried) and are not the same. (B) An electron density map calculated with phases from the 4.5 Å resolution structure of the BldD-(c-di-GMP)-21-mer complex and contoured at 1σ. (C) Two views of the crystal structure of the *S. venezuelae* FL BldD-(c-di-GMP)-DNA complex. One BldD subunit is red and the other green. The c-di-GMP molecules are shown as sticks and surfaces and colored magenta. The DNA is shown as a yellow cartoon. The DNA forms a pseudocontinuous helix in the crystal, which stabilizes the DBD-DNA interaction while the c-di-GMP dimerized CTDs are flexibly tethered to their DBDs. One of the CTDs (green) makes a weak interaction with its DBD while the other CTD (red) is fastened in place in the crystal via contacts to a symmetry-related CTD. (D) Proteolysis of FL *S. venezuelae* BldD by endoprotease Glu-C. Lane 1: BldD-(c-di-GMP)-DNA, Lane 2: BldD-DNA, Lane 3: apo FL BldD. Neither the presence of DNA nor c-di-GMP protects the linker region between the DBD and CTD from proteolysis.

Dimeric BldD binds pseudo-palindromic DNA sites that contain a 5′-TNAC(N)_5_GTNA-3′ consensus (den Hengst et al., 2010). Our data and those of others (Lee et al., 2007a) show that the BldD DBD alone can dimerize at higher concentrations (≥10 μM). Consistent with these findings, the crystal structure of the *S. coelicolor* BldD DBD revealed a dimer with a small contact interface (Kim et al., 2006). We obtained additional views of the BldD DBD by solving the *S. venezuelae* BldD DBD structure to 2.80 Å resolution. This structure contained two DBD dimers in the asymmetric unit. Comparison of these dimers with the dimer from the *S. coelicolor* DBD structure showed that, although hydrophobic residues within the C-terminal regions of the DBDs make contacts between the subunits in each case, all three dimers take distinct conformations (Figure S6A). Moreover, there is less than 300 Å^2^ BSA per subunit in this dimer, which is far less than the 1000 Å^2^ BSA per subunit typically observed for biologically relevant dimers (Krissinel and Henrick, 2007). These data indicate that the BldD DBD is unlikely to form a stable DNA-binding active dimer at physiologically relevant concentrations. In addition, although the BldD DBD resembles the equivalent DBD of λ repressor, previous modeling studies suggested that the BldD DBDs would not interact specifically with DNA if BldD employed a DNA-binding mechanism similar to that utilized by λ repressor (Kim et al., 2006). Thus, it has been unclear how BldD binds cognate DNA. Our finding that c-di-GMP binding leads to the formation of a c-di-GMP bridged CTD dimer provides the missing link to this puzzle. However, how c-di-GMP binding to the CTDs is signaled to the DBDs to bring about DNA binding remained unclear.

**Figure 7:**
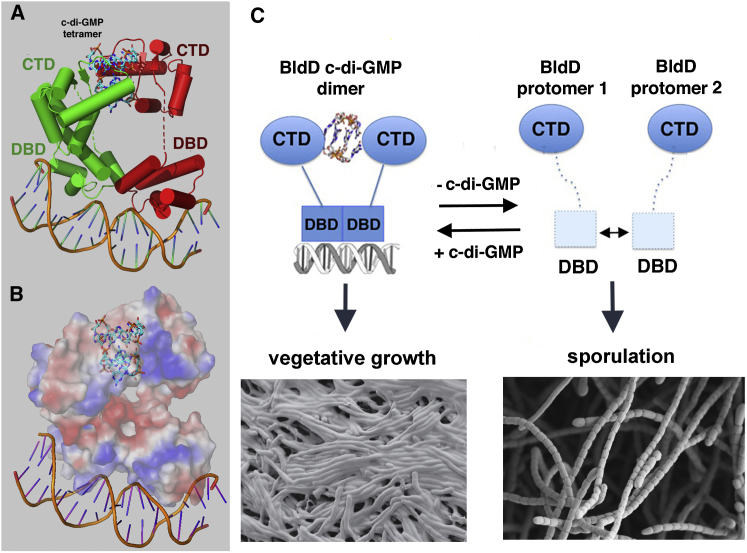
The Molecular Mechanism of c-di-GMP-Activated DNA Binding by BldD and Its Control of *Streptomyces* Development (A) Structure of the *S. venezuelae* BldD-(c-di-GMP)-DNA complex. One protomer is colored red and the other green. The c-di-GMP molecules are shown as sticks and the DNA as a cartoon. The linker region between the DBD and CTD (red or green dashed lines) is disordered in both protomers, indicating their conformational flexibility. (B) Electrostatic surface representation of the BldD-(c-di-GMP)-DNA structure shown in the same orientation as (A). (C) Schematic model of c-di-GMP-mediated activation of high affinity DNA binding by BldD, leading to repression of *Streptomyces* development. The BldD CTD is shown as ovals and the DBD as squares. The DBDs interact only weakly in vivo (indicated by the double-headed arrow). Increased c-di-GMP levels lead to BldD CTD dimerization, resulting in a significant increase in the local concentration of the DBDs, allowing them to dimerize optimally in the presence of cognate DNA to effect high affinity DNA binding. This leads to repression of the BldD regulon, thus blocking multicellular differentiation. See also Figure S6 and Table S2.

To deduce the mechanism by which c-di-GMP activates BldD to bind DNA, we determined the structure of a BldD-(c-di-GMP)-21-mer DNA complex to 4.5 Å resolution (Extended Experimental Procedures; Figure S6B). While the low resolution of the structure precludes a detailed analysis, the electron density maps show the overall arrangement of the domains and how the DBDs dock onto the DNA. Critically, the structure reveals that BldD binding to cognate DNA is more similar to the DNA binding mode of the XRE protein SinR (Lewis et al., 1998, Newman et al., 2013) than to that of the λ repressor, as the two BldD DBDs are juxtaposed when BldD is bound to DNA (Figures 7A and 7B). The DBD-DBD interacting surfaces observed in the BldD-DNA complex correspond to the hydrophobic regions near the DBD C terminus that interact in the apo DBD structures. However, the interfaces are yet again different, supporting the notion that DBD dimerization is weak and malleable. Such malleability is critical to allow the dimeric BldD HTH elements to bind the DNA, which is bent by ∼30°.

The BldD DBDs are tethered to the CTD via a linker that was previously shown to be highly flexible (Kim et al., 2006, Lee et al., 2007a). Not surprisingly, this linker (PGTTPGGAAEPPP; residues 71–84) is disordered in the BldD-(c-di-GMP)-21-mer structure. Its flexibility is underscored by the different orientation of the two CTDs in the structure relative to the DBD-DNA complex (Figure 7A). The CTD subunits in the dimer make different interactions that help anchor them in the BldD-(c-di-GMP)-DNA crystal; one of the CTDs interacts with a hydrophobic patch on its cognate DBD, while the other CTD makes crystal contacts with a symmetry mate (Figures 7A and S6C). However, the CTDs in the FL BldD-(c-di-GMP)-DNA structure are dimerized in a manner identical to that observed in our CTD-(c-di-GMP) structures (Figures 4D, 5A, 7A, and S6C).

Additional evidence that the linker region between the DBD and CTD is flexible was provided by proteolysis experiments employing Endoproteinase Glu-C, which cleaves exposed peptide bonds on the carboxyl side of glutamic or aspartic acid residues. Hence, if the BldD linker region is unstructured, Glu-C should selectively cleave after BldD residue Glu80. Glu-C proteolysis experiments were carried out on the FL BldD protein, the FL protein with the 21-mer DNA present, and the FL protein in the presence of both c-di-GMP and the 21-mer DNA. In all three cases BldD was readily cleaved into two bands, corresponding to the DBD and the CTD (Figure S6D). Thus, neither the presence of DNA, nor of c-di-GMP and DNA, protected BldD from proteolysis, indicating the linker is exposed even in the presence of cognate ligands. Therefore, the combined data suggest a molecular model for c-di-GMP activation of BldD DNA binding in which binding of c-di-GMP leads to the formation of a c-di-GMP-linked BldD CTD dimer (Figure 7C). Such CTD dimerization effectively brings the two DBDs into proximity, thereby increasing their local concentration to allow germane DBD dimerization on cognate DNA (Figure 7C). The inherent flexibility afforded by the DBD-CTD linker allows the DBDs to adjust for optimal binding to multiple pseudo-palindromic BldD DNA boxes.

## Discussion

The role of c-di-GMP has been studied extensively in unicellular Gram-negative bacteria, in which most c-di-GMP-dependent signaling pathways control the transition from a planktonic, motile lifestyle to a surface-associated, sessile lifestyle (“stick or swim”). Here we show that the activity of the *Streptomyces* master regulator BldD is also controlled by c-di-GMP, thus bringing the regulatory role of this key second messenger into a new physiological arena, that of differentiation in Gram-positive multicellular bacteria. Our studies indicate that c-di-GMP binding to BldD controls the developmental switch between vegetative growth and sporulation. Specifically, we demonstrate that c-di-GMP binding to BldD activates its DNA-binding activity, which results in repression of sporulation genes during vegetative growth. Consistent with this, genetic studies revealed that *bldD* null mutants sporulate precociously, mimicking the effect of overexpressing a c-di-GMP phosphodiesterase. Thus, c-di-GMP signals through BldD to control the hypha-to-spore developmental transition in *Streptomyces*.

Few c-di-GMP effector-binding motifs have been identified to date. These include I-site motifs (Duerig et al., 2009, Lee et al., 2007b, Petters et al., 2012), inactive EAL domains (Navarro et al., 2009, Qi et al., 2011, Newell et al., 2009), and PilZ domains (Amikam and Galperin, 2006). Structures have shown that c-di-GMP interacts with these motifs as either a monomer or intercalated dimer. The BldD protein does not contain any previously characterized c-di-GMP effector-binding motifs. Thus, to elucidate the mechanism by which c-di-GMP acts as a switch to turn on the DNA-binding activity of BldD, we determined several structures of the BldD CTD complexed to c-di-GMP as well as a 4.5 Å structure of the BldD-(c-di-GMP)-DNA complex. These structures revealed that BldD interacts with c-di-GMP using a heretofore unseen c-di-GMP binding mode involving a unique c-di-GMP-binding signature sequence consisting of two proximal arginine and aspartic acid containing motifs, motif 1 (RXD) and motif 2 (RXXD), separated by eight residues. Remarkably, in this binding mode, a tetrameric form of the c-di-GMP functions as a small-molecule dimerizer to adjoin two noninteracting BldD protomers. Notably, the identical CTD dimer-(c-di-GMP) tetramer structure was seen in multiple crystal forms. Finally, binding studies confirmed that the BldD CTD binds c-di-GMP with a stoichiometry of four c-di-GMP molecules to one BldD CTD dimer. The c-di-GMP tetramer revealed in these structures represents a previously unknown form of this nucleotide second messenger. Indeed, we do not know of any other example in which a signaling molecule can assume different oligomeric states to effect its function.

**Figure S7:**
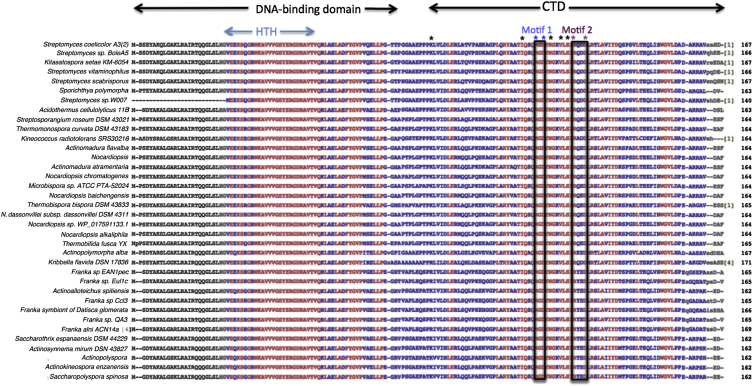
Multiple Sequence Alignment Resulting from BLAST of the *S. coelicolor* BldD Protein, Related to Figure 5 Proteins that were > 95% identical were not included. Non-conserved residues are colored gray, highly conserved residues are blue and residues that are identical are colored red. *S. coelicolor* and *S. venezuelae* BldD share 100% identity within their DNA-binding domains; an alignment of their CTD sequences is shown in Figure 5A. The residues encompassing the DNA-binding domain and CTD are indicated as are the regions that correspond to the helix-turn-helix DNA-binding motif and Motifs 1 and 2 (blue and magenta) in the CTD. Key residues in Motif 1 (R114 and D116) and Motif 2 (R125 and D128) that contact the c-di-GMP are indicated by blue and magenta asterisks, respectively, and are completely conserved among the CTDs of all BldD proteins. Other residues that contact c-di-GMP are indicated by black asterisks. Such strong conservation within these actinomycetes suggests that BldD regulation via c-di-GMP is conserved across these organisms. The sequence alignment is better viewed at 300%.

BldD is present throughout the sporulating actinomycetes (den Hengst et al., 2010), including, for example, nitrogen-fixing *Frankia* that live in symbiosis within the root nodules of alder trees, and members of the marine genus *Salinospora*, which have recently emerged as an important source of antibiotics and other medically significant compounds. Outside of the genus *Streptomyces*, the only actinomycete in which BldD has been investigated is *Saccharopolyspora erythraea*, where BldD directly controls expression of the biosynthetic cluster of the clinically important antibiotic erythromycin (Chng et al., 2008). Homologs from across the sporulating actinomycetes share 77%–99% sequence identity with *S. venezuelae* BldD. The main region of conservation between these proteins is the N-terminal DNA-binding domain, which shares 95%–100% identity. Although our BldD-(c-di-GMP)-DNA structure is too low resolution to ascribe specific protein-DNA contacts, it reveals the location of the HTH motif and residues that likely contact the DNA. Notably, these amino acids are the most conserved among BldD homologs (essentially 100%; Figure S7). By contrast, the CTD regions of BldD proteins are less well conserved (as low as 48% identity). Hence, it is striking, given this low conservation, that the residues that interact with c-di-GMP are strictly conserved (Figure S7). The only exception is Lys84, which contacts c-di-GMP phosphate groups. However, in all BldD homologs this residue is either a lysine or arginine and thus able to make the same electrostatic interaction. Of particular note, residues R114, D116, R125, and D128 (from motifs 1 and 2), which mediate essential specifying contacts with c-di-GMP, are conserved in all homologs (Figure S7). Further, all of the actinomycetes that encode an ortholog of BldD also encode GGDEF domain-containing DGCs. These combined findings indicate that BldD-(c-di-GMP) is likely to control key developmental processes throughout the sporulating actinomycetes, using tetrameric c-di-GMP as a second messenger.

## Experimental Procedures

For a full explanation of the experimental protocols, see Extended Experimental Procedures in Supplemental Information.

### Bacterial Strains, Plasmids, and *bldD* Null Mutant Construction

Strains and plasmids used are shown in Table S3, and oligonucleotides used are shown in Table S4. Plasmids were constructed as described in Extended Experimental Procedures. A *bldD* null mutant (SV77) was constructed by Redirect PCR targeting, and the *bldD::apr* mutant allele was moved into a new WT background by generalized transduction using the *S. venezuelae*-specific phage SV1. Transduction of the *bldD::apr* allele was confirmed by PCR and the strain was named SV74.

### Capture of c-di-GMP-Binding Proteins and Differential Radial Capillary Action of Ligand Assays

Cyclic di-GMP capture compound experiments were performed as described previously (Nesper et al., 2012) but with the minor modifications described in Extended Experimental Procedures. Briefly, the c-di-GMP capture compound was added to the soluble lysates and, following UV irradiation in the caproBox, magnetic streptavidin beads were added to the reaction. After incubation the beads were collected, washed, and boiled in sample buffer, the proteins released were run on SDS-PA gels and cut out for mass spectrometry analysis. The DRaCALA assays used His_6_-BldD or N-terminally His-tagged domains, which were incubated with ∼11 nM ^32^P-c-di-GMP. The competition experiments had 266 μM cold c-di-GMP or GTP added to the reaction. Samples were spotted onto nitrocellulose membranes and analyzed using Phosphorimaging.

### DNA-Binding Assays: EMSA and ChIP-Seq Experiments

DNA fragments spanning the *bldM* (158 bp) and *whiG* (151 bp) promoter regions of *S. venezuelae* were generated by PCR and 5′ end labeled using [γ^32^-P]-ATP and T4 polynucleotide kinase. The binding reactions were performed using 0.6 μM His_6_-BldD and radiolabeled DNA (∼8,000 cpm) as well as 0.5 μg poly[d(I-C)] as nonspecific competitor DNA. When appropriate, increasing amounts of c-di-GMP (0.25–1.75 μM) were added to the mixture. The reaction samples were incubated for 20 min at room temperature and then run on 5% polyacrylamide gels. Chromatin immunoprecipitations were performed as described (Bush et al., 2013) using an anti-BldD polyclonal antibody.

### Biochemical Studies on BldD Oligomeric State

The oligomeric states of BldD and its domains were analyzed via chemical crosslinking using DSS in the presence and absence of c-di-GMP (see the Extended Experimental Procedures) and visualized on SDS-PA gels. Molecular weight analyses using SEC experiments were performed with a HiLoad 16/600 Superdex 75 pg column.

### Crystallization and Structures Determination of BldD Complexes

For detailed descriptions of the protein expression, purification, crystallization, structure determination, and refinement protocols, see the Extended Experimental Procedures.

Extended Experimental Procedures **Bacterial Strains, Growth Conditions, and Conjugations**

All *E. coli* strains used in this study (Table S3) were grown in LB medium under aeration at 37°C. *E. coli* DH5α was used for plasmid and cosmid propagation and BL21(DE3)pLysS for protein overexpression. BW25113 (Datsenko and Wanner, 2000) containing a λ RED plasmid, pIJ790, was used to create the *bldD* disruption cosmid and ET12567 containing pUZ8002 (Paget et al., 1999) was used for conjugation experiments. *S. venezuelae* strains (Table S3) were grown at 30°C on maltose-yeast extract-malt extract (MYM) medium (Stuttard, 1982) containing 50% tap water (MYM-TAP) and 200 μl trace element solution (Kieser et al., 2000) per 100 ml. Liquid cultures were grown under aeration at 250 rpm. Conjugations between *E. coli* and *S. venezuelae* were carried out as previously described (Bibb et al., 2012).

**Construction of Plasmids**

The oligonucleotides used for plasmid constructions are listed in Table S4. For protein overexpression and purification, *bldD* and its individual domains were cloned into pET15b resulting in N-terminally His-tagged FL (Full-length) BldD (amino acid residues 1-166), BldD-DBD (amino acid residues 1-79) and BldD-CTD (amino acid residues 80-166). For overexpression of *yhjH* in *S. venezuelae*, an N-terminally codon optimized variant of *yhjH* was cloned downstream of the *ermEp*^∗^ promoter in the ΦBT1 *attB* site-specific integrative vector pIJ10257 (Hong et al., 2005). Point mutations in *bldD* were introduced by following the four-primer/two-step PCR protocol (Germer et al., 2001). For complementation analysis the *bldD* gene carrying its native promoter and the R114D, D116R, R125D and D128R mutations was expressed from the integrative vector pMS82.

**Construction of a *bldD* Null Mutant Derivative of *S. venezuelae* and Phage Transduction**

The *bldD* mutant was generated according to the Redirect PCR targeting protocol (Gust et al., 2003, Gust et al., 2004). The Sv-4-H05 cosmid was introduced into *E. coli* BW25113 and *bldD* was replaced with the apramycin-resistance (*apr*) cassette containing *oriT*, which was amplified from pIJ773 using primers with *bldD*-specific extensions (Table S4). The disrupted cosmid was confirmed by restriction and PCR analyses and introduced into *E. coli* ET12567/pUZ8002 for conjugation into *S. venezuelae*. A null mutant generated by double crossing over was identified by its apramycin-resistant and kanamycin-sensitive phenotype and named SV77 after confirmation by PCR using test primers listed in Table S4. The mutant allele *bldD::apr* was moved into a new WT background by generalized transduction using the *S. venezuelae*-specific phage SV1 (Stuttard, 1979). To prepare SV1-lysate, 10^4^ phage were added to 10^6^ SV77 donor spores in 800 μl pre-warmed (45°C) soft nutrient agar (SNA) and poured onto Difco nutrient agar plates containing 0.5% glucose, 10 mM MgSO_4_ and 10 mM Ca(NO_3_)_2_. The plates were incubated at 30°C overnight, then flooded with 2.5 ml Difco nutrient broth (DNB) and incubated for 3-4 hr at room temperature. The phage-containing DNB soak-out was harvested and filtered through a 0.45 μm filter to eliminate bacterial contamination. For transduction of the *bldD::apr* allele, 10^9^ phage particles harvested from the *bldD::apr* mutant strain SV77, were mixed with 10^7^-10^8^ WT spores and incubated overnight on MYM agar at room temperature before overlaying with apramycin for selection. Plates spread with the recipient strain or the phage alone were used as controls. Transduction of the *bldD::apr* allele was confirmed by PCR using test primers listed in Table S4, and the strain was named SV74.

**c-di-GMP Protein Capture Experiments**

Cyclic di-GMP capture compound experiments were performed as described previously (Nesper et al., 2012) with minor modifications. *S. venezuelae* cultures were grown in MYM-TAP supplemented with trace element solution until late transition phase for 24 hr at 30°C and then pelleted by centrifugation for 10 min at 6,000 rpm. The pellet was resuspended in lysis buffer (6.7 mM MES, 6.7 mM HEPES, 200 mM NaCl, 6.7 mM potassium acetate (KAc), pH 7.5) containing protease inhibitor and DNase I. Cells were put through a French Press four times at 18,000 psi and then centrifuged at 100,000 x g for 1 hr. For the capture experiments, the protein concentration of the soluble fraction was determined using a UV/Vis Nanodrop spectrophotometer and 400 μg protein were mixed with 10 μM c-di-GMP capture compound and with 20 μl 5 x capture buffer (100 mM HEPES, 250 mM KAc, 50 mM magnesium acetate (MgAc), 50% glycerol, pH 7.5). 1 mM c-di-GMP was added to the control reaction and incubated for 30 min prior to capture compound addition. The reaction volume was adjusted with H_2_O to 100 μl and incubated for 2 hr at 4°C in the dark on a rotary wheel. After UV irradiation for 4 min in a caproBox, 50 μl magnetic streptavidin beads and 25 μl 5 x wash buffer (250 mM Tris pH 7.5, 5 M NaCl, 0.1% n-octyl-β-glucopyranoside) were added to the reaction and the mixture was incubated for 45 min at 4°C on a rotary wheel. The beads were then collected with a magnet and washed 6 times with 200 μl wash buffer. The beads were resuspended in 20 μl sample buffer and run for ∼10 min on a 15% SDS polyacrylamide gel after 10 min incubation at 95°C. The gel was stained using InstantBlue Coomassie Stain solution, and a 1×1 cm gel slice containing all captured proteins was excised for analysis by mass spectrometry.

**Electrophoretic Mobility Shift Assays**

DNA fragments spanning the *bldM* (158 bp) and *whiG* (151 bp) promoter regions of *S. venezuelae* were generated by PCR using oligonucleotides listed in Table S4 and then 5′ end-labeled using [γ^32^-P]-ATP and T4 polynucleotide kinase. The binding reactions were performed in bandshift buffer (10 mM Tris pH 7.5, 1 mM EDTA, 5% glycerol, 10 mM NaCl, 1 mM MgCl_2_) in 20 μl reaction mixture containing 0.6 μM BldD and radiolabeled DNA (∼8,000 c.p.m.) as well as 0.5 μg poly[d(I-C)] as nonspecific competitor DNA. When appropriate, increasing amounts of c-di-GMP (0.25 – 1.75 μM) were added to the mixture. The reaction samples were incubated for 20 min at room temperature followed by electrophoresis on a 5% polyacrylamide gel in 0.5 x TBE (Tris-Borate-EDTA) buffer at 80V for 105 min. The gels were dried before being analyzed on a Phosphorimager.

**BldD ChIP-Seq Experiments**

*S. venezuelae* strains for ChIP-seq were grown in MYM-TAP. Chromatin immunoprecipitations were performed as previously described (Bush et al., 2013), except that an anti-BldD polyclonal antibody was used and pulled down with protein A-sepharose beads. Library construction, sequencing and ChIP-seq data analyses were all carried out as previously described (Bush et al., 2013).

**Chemical Crosslinking and SDS Polyacrylamide Gel Electrophoresis**

The His_6_-BldD-DBD and His_6_-BldD-CTD proteins were dialyzed into crosslinking buffer (100 mM NaH_2_PO_4_, 150 mM NaCl, pH 8) and then incubated at room temperature for 30 min in 20 μl reaction samples containing 10 μM protein, 1 mM disuccinimidyl suberate (DSS) in dimethylsulfoxide (DMSO), and c-di-GMP as indicated. The reaction was stopped by adding 50 mM Tris pH 8 and incubation for 15 min followed by addition of SDS sample buffer and heating to 95°C for 10 min. Samples were separated on a 15% SDS polyacrylamide gel and visualized by Coomassie staining.

**Determination of c-di-GMP Binding to Proteins by Differential Radial Capillary Action of Ligand Assay**

Radiolabeled c-di-GMP was synthesized in vitro using [γ^32^-P]-GTP and the purified diguanylate cyclase PleD^∗^ as described (Paul et al., 2004). The DRaCALA assays (Roelofs et al., 2011) were performed using 2 μg of His_6_-BldD or its N-terminally His-tagged domains that were incubated with ∼11 nM ^32^P-c-di-GMP in DGC buffer (250 mM NaCl, 25 mM Tris pH 8, 10 mM MgCl_2_, 5 mM β-mercaptoethanol). For competition experiments, 266 μM cold c-di-GMP or GTP were added to the reaction. After a 5 min incubation at room temperature, 5 μl of the binding sample were spotted onto nitrocellulose membrane and the dried membranes were analyzed using a Phosphorimager.

**Purification, Crystallization, and Structure Determination of *S. venezuelae* and *S. coelicolor* BldD CTD-(c-di-GMP) Complexes**

For structural studies on the CTD, the regions encoding residues 80-166 (*S. venezuelae* BldD) and 80-167 (*S. coelicolor* BldD) were cloned into the pET15b vector and the proteins induced at 37°C and purified via Ni-NTA chromatography. The His-tags were removed from the proteins used for structural studies by thrombin cleavage. Crystals of the *S. venezuelae* BldD CTD-(c-di-GMP) complex, which assumed the trigonal space group, P3_2_, were obtained using protein at 30 mg/mL and 1 mM c-di-GMP. Crystals were produced via the hanging drop vapor diffusion method and mixing the complex 1:1 with 28% PEG 1500, 100 mM sodium acetate, pH 5.5. The *S. venezuelae* BldD CTD contains no methionines and hence for phasing, Leu92 and Ile135 were substituted with methionines. Semet(L92M/I135M) BldD CTD was expressed using the methionine inhibitory pathway and the protein purified and crystallized with c-di-GMP as per the WT CTD. The selenomethionine-substituted L92M/I135M protein crystallized in the wild-type protein P3_2_ space group. A second crystal form of the *S. venezuelae* BldD CTD-(c-di-GMP) complex was grown with protein at 20-40 mg/mL and 1 mM c-di-GMP using 20% PEG 2000 monomethyl ether, 100 mM MES, pH 6.0, as a crystallization reagent and took the orthorhombic space group P2_1_2_1_2. The third crystal form of the *S. venezuelae* BldD CTD-(c-di-GMP) complex was produced with protein at 10 mg/mL and 1 mM c-di-GMP using 1.2 M sodium/potassium phosphate, 50 mM citrate pH 5.6. These crystals take the orthorhombic space group C222_1_. Crystals were obtained for the *S. coelicolor* BldD CTD-(c-di-GMP) complex using 25 mg/mL protein, 1 mM c-di-GMP and mixing the complex 1:1 with a reservoir comprised of 1.4 M sodium/potassium phosphate, 100 mM HEPES pH 7.5. These crystals take the C222_1_ space group.

Multiple wavelength anomalous diffraction (MAD) data were collected for a Semet(L92M/I135M) *S. venezuelae* BldD CTD-(c-di-GMP) crystal to 2.28 Å resolution at ALS (Advanced Light Source, Berkeley, CA, USA) beamline 8.3.1 (Table S1). The data were processed using MOSFLM and the heavy atom substructure was obtained via SOLVE (Terwilliger and Berendzen, 1999). The figure of merit (FOM) for the solution was 0.65. Phenix was used for final phasing and density modification (Adams et al., 2010). The crystal contains 12 protein molecules in the asymmetric unit (ASU) and each of the six dimers is glued together by four c-di-GMP molecules. The six c-di-GMP complexed dimers are essentially identical (Figure 4D). Final refinement was done using a data set collected to 1.95 Å resolution for a Semet(BldDL92M) CTD-(c-di-GMP) crystal. A WT data set was also collected to 2.2 Å resolution and the structure was identical to the L92M and L92M/I135M structures. The final 1.95 Å resolution-structure contains residues 84-161 for each of the 12 subunits and 24 c-di-GMP molecules (Table S2). Data were collected to 1.75 Å, 2.25 Å and 2.33 Å resolution for the *S. venezuelae* and *S. coelicolor* BldD CTD-(c-di-GMP) C222_1_ forms and the *S. venezuelae* BldD CTD-(c-di-GMP) P2_1_2_1_2 crystal form, respectively, and the structures solved by molecular replacement (MR). The *S. coelicolor* BldD CTD and *S. venezuelae* BldD CTD C222_1_ crystal forms contain a CTD dimer and four c-di-GMP molecules in the ASU and the *S. venezuelae* BldD C-domain-(c-di-GMP) P2_1_2_1_2 crystal form contains 10 subunits (five dimers), and 20 c-di-GMP molecules. The structures were solved by MR using the program Phaser (McCoy et al., 2007). Final refinement statistics are provided in Table S2. The topology of every BldD CTD is β1 (residues 84-88)-α1 (residues 89-93)-α2 (residues 98-113)-β2 (residues 120-124)-α3 (residues 128-136)-α4 (residues 140-149)-α5 (residues 154-160).

**Crystallization and Structure Determination of the *S. venezuelae* BldD DBD**

The BldD DNA binding domain (BldD DBD), encoding residues 1-79, was cloned into pET15b, expressed in *E. coli* BL21(DE3) and the protein purified via Ni-NTA chromatography. Prior to crystallization, the hexa-His tag was removed via thrombin cleavage and the protein further purified by size exclusion chromatography. Crystals were grown by mixing the protein (40 mg/mL) 1:1 with a reservoir consisting of 35% PEG 400, 0.1 M MgCl_2_ and 0.1 M Tris pH 7.5. The crystals take the hexagonal space group P6_1_22. X-ray intensity data were collected to 2.8 Å resolution at ALS beamline 8.3.1 and processed with MOSFLM. The R_sym_ and I/σ(I) for the data are 11.3% (38.4%) and 11.4 (4.3), respectively, where the values in parentheses indicate data from the highest resolution shell. The structure, which contains 3 subunits in the ASU (2 subunits form a dimer and crystal symmetry generates a second dimer), was solved with Phaser using a single *S. coelicolor* DBD subunit (PDB code 2EWT) as the search model. The final model contains residues 3-71 of each subunit and was refined using Phenix to final R_work_/R_free_ values of 23.7%/28.9%, respectively (Adams et al., 2010).

**Crystallization and Structure Determination of the *S. venezuelae* BldD-(c-di-GMP)-21-Mer Complex**

Crystals of the Full-Length (FL) *S. venezuelae* BldD-(c-di-GMP)-21-mer complex were grown by using protein in which the N-terminal hexa-His tag had been cleaved and incubated with 1 mM c-di-GMP (final concentration). This BldD-(c-di-GMP) solution was mixed in a 1:1 molar ratio of BldD dimer to 21-mer DNA duplex (5′-CCCCTCACGCTGCGTGACGGG-3′, with the canonical BldD box underlined, annealed to its complementary oligodeoxynucleotide) for crystallization trials. Crystals were grown by mixing the protein-DNA complex 1:1 with the crystallization solution composed of 100 mM sodium citrate tribasic/citric acid pH 4.0 and 200 mM ammonium sulfate. The crystals take the trigonal space group, P3_2_21, with a = b = 114.0 Å, c = 95.2 Å and contain a BldD dimer-(c-di-GMP)-21-mer duplex in the ASU. Data were collected to the limiting resolution of 4.5 Å. The R_sym_ = 9.0% (86.0%) and I/σ(I) = 5.8 (1.8), where the values in parentheses are for the highest resolution shell. The structure was solved by MR in stages. First, BldD DNA binding domain-DNA complex models were constructed based on the SinR-DNA (PDB code 3ZKC) or λ repressor-DNA (PDB code 1LMB) complex structures and used in the MR program Phaser (McCoy et al., 2007). A clear solution was obtained for the SinR-DNA based model. Packing revealed that the DNA forms a pseudocontinuous helix in the crystal. This solution was then used as a static model with the CTD-(c-di-GMP) dimeric structure as a search model. A clear solution was obtained with MolRep. Due to its low resolution, the structure was subjected to rigid body refinement only (R_work_/R_free_ = 33.0%/38.9%, respectively).

**Proteolysis of FL BldD by Endoproteinase Glu-C**

To examine the flexibility of the linker region that connects the BldD DBD and CTD, limited proteolysis experiments were carried out. Specifically, the accessibility of residue Glu80, which is the only acidic residue in the BldD linker region, was determined by the ability of the Glu-C protease to cleave after this residue. In these experiments, Endo-Glu-C (100 Units/mL) was a diluted 50 fold into samples of 1) FL BldD (1 mg/mL), 2) FL BldD-21-mer DNA (1 mg/ml protein with 100 μM DNA) or 3) FL BldD-(c-di-GMP)-21-mer (1 mg/mL protein with 100 μM 21-mer DNA and 1 mM c-di-GMP). The proteolysis buffer was 50 mM sodium phosphate, pH 7.5 and the final protein concentration of each sample was 4 mg/mL. The samples shown in Figure S6D were taken at a time point of 4 hr. Notably, proteolysis of each sample was identical, demonstrating that ligand binding of neither cognate DNA nor c-di-GMP by BldD affect the accessibility of the linker region, which remains flexible and unstructured.

**Size Exclusion Chromatographic Analyses of Wild-Type BldD CTD and the BldD CTD Quadruple Mutant**

Size exclusion chromatography experiments were carried out using a HiLoad 16/600 Superdex 75 pg column. 5 mg of either the wild-type BldD CTD or the quadruple mutant were loaded onto a column that had been pre-equilibrated with 150 mM NaCl, 5% glycerol, 20 mM Tris HCl pH 7.5 and 3 μM c-di-GMP. The samples were run and eluted with the same c-di-GMP containing buffer. The elution volume was plotted against a standard curve to determine the relative molecular weights of the samples. The standard curve was determined using cytochrome C (12 kDa), carbonic anhydrase (29 kDa) and albumin (66 kDa).

**Cryo-Scanning Electron Microscopy**

Cryo-SEM was performed as previously described (Bush et al., 2013).

**BldD CTD Expression and Purification from Sf9 Cells**

When induced in *E. coli* BL21(DE3) at 37°C, BldD CTD samples contained little to no c-di-GMP contamination, which was supported by A_280_/A_260_ values of the purified protein, which were approximately 1.6. However, to ensure that there was no endogenous c-di-GMP present in samples used for binding affinity measurements, the BldD CTD was expressed and purified in Sf9 insect cells. For these studies, a gene encoding the same *S. venezuelae* BldD CTD region that was expressed in *E. coli* was codon optimized for expression in insect cells (Genscript, Piscataway, NJ, USA; http://www.genscript.com) and subcloned into the expression vector F1, which was transfected into the DH10Bac strain for generation of the recombinant bacmid. Positive *bldD CTD* containing clones were identified by PCR. rBacmids were then transfected in Sf9 insect cells with Cellfectin II and the cells incubated in Sf-900 II SFM for 56 days before harvest. The supernatant was collected for the P1 viral stock and P2 was amplified for later infection. Sf9 cells expressing BldD CTD were harvested at 72 hr post infection and cells were lysed into 25 mM Tris pH 7.5, 300 mM NaCl and 5% glycerol with protease inhibitors. The protein was purified from the supernatant by Ni-NTA followed by size exclusion chromatography and was > 90% pure as assessed by SDS-PAGE analysis.

**Determination of the Affinity, Stoichiometry, and Specificity of c-di-GMP for Sf9-Purified BldD CTD by Fluorescence Polarization**

To measure binding, 2′-O-(6-[Fluoresceinyl]aminohexylcarbamoyl)-cyclic diguanosine monophosphate (2′-Fluo-AHC-c-di-GMP), was used as the fluoresceinated ligand. This molecule is conjugated via a 9 atom spacer to one of the 2′ hydroxyl groups of the c-di-GMP, hence meeting the structural requirement for BldD binding that only one 2′ hydroxyl group be unmodified and the other available for the interactions observed in the BldD-(c-di-GMP) structures (Figure 5). Binding was carried out at 25°C in a buffer of 150 mM NaCl and 25 mM Tris-HCl pH 7.5, which contained 1 nM 2′-Fluo-AHC-c-di-GMP. Increasing concentrations of BldD CTD were titrated into the reaction mixture to obtain the binding isotherms. After each addition of protein the reaction sample was incubated for 30 min to ensure equilibrium had been reached. c-di-GMP binding to BldD appears to have the characteristics of a high affinity/slow binding ligand. The resulting data were plotted using KaleidaGraph and curves were fitted to deduce binding affinities. To determine the binding stoichiometry of the BldD CTD-(c-di-GMP) complex, the same FP binding conditions were used but the total concentration of c-di-GMP (c-di-GMP + 2′-Fluo-AHC-c-di-GMP) was 25 μM, ∼10-fold above the K_d_ of 2.5 μM ensuring stoichiometric binding. Close inspection of the binding isotherm revealed the possibility that there are two nearly identical binding events (likely corresponding to the binding of each intercalated c-di-GMP dimer). The graph of the resulting data shows a linear increase in the observed mPs until saturation of the binding sites, after which the line is flat. The inflection point(s) are shown in Figure S5C. Importantly, the final inflection occurs at a BldD monomer concentrations of 12 μM, which, when divided by the concentrations of c-di-GMP (25 μM), indicates a stoichiometry of two CTD protomers per four c-di-GMPs. As anticipated from a purely chemical complementarity-orientated argument, FP studies carried out in the same buffer also revealed that wild-type BldD CTD does not bind to c-di-AMP. In addition, the BldD DGR-X_8_-DQDR CTD mutant did not bind the c-di-GMP probe.

## Author Contributions

N.T. designed, performed, and interpreted experiments, created figures, and wrote the paper. M.A.S. designed, performed, and interpreted experiments, created figures, and wrote the paper. S.S. designed, performed, and interpreted experiments and created figures. N.B.C. performed experiments. K.C.F. performed experiments. R.G.B. designed and interpreted experiments and wrote the paper. M.J.B. designed and interpreted experiments and wrote the paper.

## Accession Numbers

The Protein Data Bank (PDB) accession numbers for the crystallographic work reported in this paper are 4OAX, 4OAY, 4OAZ, and 4OB4, and the ArrayExpress accession number for the ChIP-seq data is E-MTAB-2196.

## Supplemental Information

Document S1. Tables S1–S4

Document S2. Article plus Supplemental Information
