# Supplementary material for: Tetrameric c-di-GMP Mediates Effective Transcription Factor Dimerization to Control Streptomyces Development
Source: Cell. 2014 Aug 28;158(5):1136–47. doi: 10.1016/j.cell.2014.07.022 (PMC4151990; doi:10.1016/j.cell.2014.07.022)
Supplement: Document S1. Tables S1–S4 [file mmc1.pdf]

Table S1. Selected MAD Statistics for $P3_2$ BldD CTD-c-di-GMP Data, Related to Figures 4 and 5

Wavelength (Å)	0.979 (Peak)	0.979 (Inflection)	0.923 (Remote)
Space group	$P3_2$	$P3_2$	$P3_2$
a, b, c (Å)	a=b=86.5, c=151.5	a=b=86.5, c=151.5	a=b=86.5, c=151.5
α, β, γ (°)	90, 90, 120	90, 90, 120	90, 90, 120
Resolution (Å)	75.4-2.28	75.4-2.28	75.4-2.28
R_{sym} (%)[a]	5.7 (18.9)[b]	4.6 (44.3)	5.0 (30.9)
$I/\sigma(I)$	15.5 (3.0)	14.2 (2.3)	13.8 (2.5)
Total reflections (#)	317916	118771	72566
Unique reflections (#)	59112	36721	9226

[a]$R_{sym} = \Sigma\Sigma|I_{hkl}-I_{hkl(j)}|/ \Sigma I_{hkl}$, where I_{hkl} is observed intensity and $I_{hkl(j)}|$ is the final average value of intensity.

[b]Values in parentheses are for the highest resolution shell.

Table S2. Selected Crystallographic Refinement Statistics for BldD CTD-c-di-GMP Structures, Related to Figures 4, 5, and 7

Complex	*S. venezuelae* BldD C-domain-(c-di-GMP) (form 1)	*S. venezuelae* BldD C-domain-(c-di-GMP) (form 2)		*S. venezuelae* BldD C-domain-(c-di-GMP) (form 3)	*S. coelicolor* BldD C-domain-(c-di-GMP)
**Data Collection**					
Space group	$P3_2$	$P2_12_12$		$C222_1$	$C222_1$
a, b, c (Å)	a=b=86.5, c=151.5	a=136.5, b=98.8, c=68.8		a=36.2, b=95.5, c=99.5	a=36.9, b=95.5, c=100.1
α, β, γ (°)	90, 90, 120	90, 90, 90		90, 90, 90	90, 90, 90
Resolution (Å)	75.8-1.95	98.8-2.33		99.6-1.75	34.6-2.25
R_{sym} (%)[a]	5.7 (57.9)[b]	4.6 (44.3)		4.1 (38.5)	5.0 (30.9)
I/σI	11.4 (1.9)	14.2 (2.3)		14.6 (2.6)	13.8 (2.5)
Total reflections (#)	322678	118771		57815	72566
Unique reflections (#)	90983	36721		17271	9226
**Refinement**					
R_{work}/R_{free} (%)[c]	19.8/24.4	22.6/26.9		19.8/21.9	22.6/25.6
**Ramachandran analysis**					
Most Favored (%/#)	91.8/752	84.3/452	92.1/117	94.4/117	
Add. Favored (%/#)	8.7/67	14.7/79	7.910	5.6/7	
Allowed (%/#)	0.0/0	0.9/5	0.0/0	0.0/0	
Outlier (%/#)	0.0/0	0.0/0	0.0/0	0.0/0	
**Rmsd**					
Bond lengths (Å)	0.020	0.017	0.008	0.017	
Bond angles (Å)	1.99	2.04	1.32	2.03	

[a]$R_{sym} = \Sigma\Sigma|I_{hkl}-I_{hkl}(j)|/ \Sigma I_{hkl}$, where I_{hkl} is observed intensity and $I_{hkl}(j)|$ is the final average value of intensity.

[b]Values in parentheses are for the highest resolution shell.

[c]$R_{work} = \Sigma||F_{obs}| - |F_{calc}||/\Sigma|F_{obs}|$ and $R_{free} = \Sigma||F_{obs}| - |F_{calc}||/\Sigma|F_{obs}|$; where all reflections belong to a test set of 5% randomly selected data.

Table S3. Strains and Plasmids Used in this Study, Related to Experimental Procedures

	Relevant genotype/comments	Source or reference
**Strains**		
S. venezuelae		
ATCC 10712	Wild type	(Bibb et al., 2012)
SV70	*attB*$_{\Phi BT1}$::pIJ10350 (*ermEp**- *cdgB)*; Hyg^R	This study
SV71	*attB*$_{\Phi BT1}$::pIJ10659 (*ermEp** - *yhjH*); Hyg^R	This study
SV77	*ΔbldD::apr*; Apr^R (Redirect)	This study
SV74	*ΔbldD::apr*; Apr^R (SV1-transduction)	This study
SVNT6	*attB*$_{\Phi BT1}$::pSVNT5 (*ermEp**- *cdgB*-G472A*)*; Hyg^R	This study
SVNT8	*attB*$_{\Phi BT1}$::pSVNT4 (*ermEp**- *yhjH*-E48A*)*; Hyg^R	This study
SVNT9	*ΔbldD::apr*; Apr^R *attB*$_{\Phi BT1}$::pIJ10350 (*ermEp**- *cdgB)*; Hyg^R	This study
SVNT12	*ΔbldD::apr*; Apr^R *attB*$_{\Phi BT1}$::pSVNT7 (pMS82-*bldD* R114D, D116R, R125D, D128R); Hyg^R	This study
E. coli		
ET12567/pUZ8002	*dam*, *dcm*, *hsd*, Kan^R, Cm^R	(Paget et al., 1999)
BW25113/pIJ790	(Δ(*araD-araB*)*567*, Δ*lacZ4787*(::*rrnB-4*), *lacIp-4000*(lacI^Q), λ-, *rpoS369*(Am), *rph-1*, Δ(*rhaD-rhaB*)568, *hsdR514*; Cm^R	(Datsenko and Wanner, 2000)
BL21(DE3)/pLysS	F- *ompT hsdS*(rB- mB-) *gal dcm* λ(DE3), Cm^R	Promega
DH5α	F$^-$ endA1 glnV44 thi-1 recA1 relA1 gyrA96 deoR nupG Φ80d*lacZ*ΔM15 Δ(*lacZYA-argF*)U169, hsdR17(r_K^- m_K^+), λ–	(Hanahan, 1985)
**Plasmids**		
pIJ773	Plasmid template for amplification of the *apr-oriT* cassette for 'Redirect' PCR-targeting	(Gust et al., 2003)
pIJ790	Modified λ RED recombination plasmid [*oriR101*] [*repA101*(ts)] *araBp-gam-be-exo*, Cm^R	(Gust et al., 2003)
pUZ8002	RP4 derivative with defective oriT, Kan^R	(Paget et al., 1999)
pET15b	T7 expression vector, Amp^R	Novagen
pIJ10257	Plasmid integrating at the ϕBT1 *attB* attachment site containing the constitutive *ermEp** promoter, Hyg^R	(Hong et al., 2005)
pIJ10350	pIJ10257 carrying *cdgB* from *S.coelicolor*	(Tran et al., 2011)
pIJ10659	pIJ10257 carrying N-terminally codon optimised *yhjH* from *E. coli*	This study
pIJ10663	pET15b-*bldD*-Full	This study
pIJ10661	pET15b-*bldD*-NTD	This study
pIJ10660	pET15b-*bldD*-CTD	This study
pIJ10674	pET15b-*bldD* R114D, D116R, R125D, D128R	This study
pIJ10676	pET15b-*bldD* R125D, D128R	This study
pIJ10677	pET15b-*bldD* R114D, D116R	This study

pIJ10668	pET15b-*bldD*-CTD L92M	This study
pIJ10678	pET15b-*bldD*-CTD L92M, I135M	This study
pSVNT4	pIJ10257 carrying N-terminally codon optimised *yhjH*-E48A from *E. coli*	This study
pSVNT5	pIJ10257 carrying *cdgB*-G472A from *S.coelicolor*	This study
pSVNT7	pMS82-*bldD* R114D, D116R, R125D, D128R	This study

Table S4. Oligonucleotides Used in this Study, Related to Experimental Procedures

Nucleotides in italics indicate restriction sites and nucleotides in bold represent mutations introduced

Oligonucleotide	Sequence
**Oligonucleotides used for cloning of *bldD*-full, *bldD*-NTD and *bldD*-CTD into pET15b**	
bldDSVfull-NdeI-fw	GGTGGT*CATATG*TCCAGCGAATACGCAAAGCAGC
bldDSVfull-BamHI-rev	CTCCTC*GGATCC*TCAGTTCTCCTCGTGGGCGACG
bldDSVntd-BamHI-rev	CTCCTC*GGATCC*TCAGGCGGCCCCGCCCGGAGTCG
bldDSVctd-NdeI-fw	GGTGGT*CATATG*GAGCCGCCGCCGAAGCTCGTCC
**Oligonucleotides used for mutagenesis of *bldD***	
bldD-L92M-fw	CTGGAGCGC**ATG**GCGCACGTC
bldD-L92M-rev	GACGTGCGC**CAT**GCGCTCCAG
bldD-I135M-fw	CTGGCCGTG**ATG**TACGACCAGTC
bldD-I135M-rev	GACTGGTCGTA**CAT**CACGGCCAG
bldD R114D, D116R fw	ATCCAGTCGCAG**GAC**GGC**CGC**TACAACGGCAAG
bldD R114D, D116R rev	CTTGCCGTTGTA**GCG**GCC**GTC**CTGCGACTGGAT
bldD R125D, D128R fw	GTGCTGTCGATC**GAC**CAGGAC**CGT**CTGCGCACCCTG
bldD R125D, D128R rev	CAGGGTGCGCAG**ACG**GTCCTG**GTC**GATCGACAGCAC
**Oligonucleotides used for cloning of *yhjH* and *cdgB* into pIJ10257**	
yhjH-NdeI-fw	GGTGGT*CATATG*ATCCGCCAGGTCATCCAGCGCATCTCCAACCCTGAAG CAAGCATCGAGAGC
yhjH-HindIII-rev	CGT*AAGCTT*TCATAGCGCCAGAACCGCCGTA
cdgBSCO-NdeI-fw	CGTGGT*CATATG*GAGACCGACTCGGAGCC
cdgBSCO-HindIII-rev	CGT*AAGCTT*TCATCCGGCGCGGCGGTGCTG
**Oligonucleotides used for generating DNA fragments for gel retardation assays**	
pbldMSV-pcr-fw	CTAGCCACAGACACCGCG
pbldMSV-pcr-rev	CGACGGTCACTCGAAAGAG
pwhiGSV-pcr-fw	GTTCGAAGATGTGGCCGAC
pwhiGSV-pcr-rev	GCGTTGCCTTGAGCCGTTC
**Oligonucleotides used for amplification of the *oriT-apr* cassette with *bldD*-specific extensions**	
bldD sense	GCGCAGCCGCATGTCGTCACAGCGTCCGGGAGCGTTATGATTCCGGGG ATCCGTCGACC
bldD disruption R2	TCTGCTGGGTCCCCGTAAGGGGTTCAGTTCTCCTCGTGGTGTAGGCTGG AGCTGCTTC

Oligonucleotides used for verification of the *bldD* mutant and for cloning into pMS82

bldDSV-HindIII-pMS82-fw	GGTGGT*AAGCTT*GAAGAAACGGACCTCCTTCTCC
bldDSV-KpnI-pMS82-rev	AGTAGT*GGTACC*GTCCGTAGACGTCACCGGCAGTCG

SUPPLEMENTAL REFERENCES

Adams, P.D. *et al*. (2010). PHENIX: a comprehensive Python-based system for macromolecular structure solution. Acta Crystallogr. D Biol. Crystallogr. *66*, 213-221.

Bibb, M.J., Domonkos, Á., Chandra, G., and Buttner, M.J. (2012). Expression of the chaplin and rodlin hydrophobic sheath proteins in *Streptomyces venezuelae* is controlled by σ^{BldN} and a cognate anti-sigma factor, RsbN. Mol. Microbiol. *84*, 1033-1049.

Bush, M.J., Bibb, M.J., Chandra, G., Findlay, K.C., and Buttner, M.J. (2013). Genes required for aerial growth, cell division and chromosome segregation are targets of WhiA before sporulation in *Streptomyces venezuelae.* mBio *4*, e00684-13.

Datsenko, K.A., and Wanner, B.L. (2000). One-step inactivation of chromosomal genes in *Escherichia coli* K-12 using PCR products. Proc. Natl. Acad. Sci. USA *97*, 6640-6645.

Germer, J., Becker, G., Metzner, M., and Hengge-Aronis, R. (2001). Role of activator site position and a distal UP-element half-site for sigma factor selectivity at a CRP/H-NS activated σ^S-dependent promoter in *Escherichia coli*. Mol. Microbiol. *41*, 705–716.

Gust, B., Challis, G.L., Fowler, K., Kieser, T., and Chater, K.F. (2003). PCR-targeted *Streptomyces* gene replacement identifies a protein domain needed for biosynthesis of the sesquiterpene soil odor geosmin. Proc. Natl. Acad. Sci. USA *100*, 1541-1546.

Gust, B., Chandra, G., Jakimowicz, D., Yuqing, T., Bruton, C., and Chater K.F. (2004). Lambda red-mediated genetic manipulation of antibiotic-producing Streptomyces. Adv. Appl. Microbiol. *54*, 107-128.

Hanahan, D. (1985). In DNA Cloning: A Practical Approach, D.M. Glover, ed. (McLean, Virginia: IRL Press), p. 109.

Hong, H.J., Hutchings, M.I., Hill, L.M., and Buttner, M.J. (2005). The role of the novel Fem protein VanK in vancomycin resistance in *Streptomyces coelicolor*. J. Biol. Chem. *280*, 13055-13061.

Kieser, T., Bibb, M.J., Buttner, M.J., Chater, K.F. and Hopwood, D.A. (2000). *Practical Streptomyces Genetics*. The John Innes Foundation, Norwich, United Kingdom.

McCoy, A.J., Crosse-Kunstleve, R.W., Adams, P.D., Winn, M.D., Storoni, L.C., and Read, R.J. (2007). Phaser crystallographic software. J. Appl. Crystallogr. *40*, 658-674.

Molle, V., Palframan, W.J., Findlay, K.C., and Buttner, M.J. (2000). WhiD and WhiB, homologous proteins required for different stages of sporulation in *Streptomyces coelicolor* A3(2). J. Bacteriol. *182*, 1286-1295.

Nesper, J., Reinders, A., Glatter, T., Schmidt, A., and Jenal, U. (2012). A novel capture compound for the identification and analysis of cyclic di-GMP binding proteins. J. Proteomics. *75*, 4874-4878.

Paget, M.S., Chamberlin, L., Atrih, A., Foster, S.J., and Buttner, M.J. (1999). Evidence that the extracytoplasmic function sigma factor σ^E is required for normal cell wall structure in *Streptomyces coelicolor* A3(2). J. Bacterial. *181*, 204-211.

Paul, R., Weiser, S., Amiot, N.C., Chan, C., Schirmer, T., Giese, B., and Jenal, U. (2004). Cell cycle-dependent dynamic localization of a bacterial response regulator with a novel di-guanylate cyclase output domain. Genes Dev. *18*, 715-727.

Pesavento, C., Becker, G., Sommerfeldt, N., Possling, A., Tschowri, N., Mehlis, A., and Hengge, R. (2008). Inverse regulatory coordination of motility and curli-mediated adhesion in *Escherichia coli*. Genes Dev. *22*, 2434-2446.

Roelofs, K.G., Wang, J., Sintim, H.O., and Lee, V.T. (2011). Differential radial capillary action of ligand assay for high-throughput detection of protein-metabolite interactions. Proc. Natl. Acad. Sci. USA *108*, 15528-33.

Stuttard, C. (1979). Transduction of auxotrophic markers in a chloramphenicol-producing strain of *Streptomyces*. J. Gen. Microbiol. *110*, 479-82.

Stuttard, C. (1982). Temperate phages of *Streptomyces venezuelae*: lysogeny and host specificity shown by phages SV1 and SV2. Microbiology 128, 115-121.

Terwilliger, T.C., and Berendzen, J. (1999). Automated MAD and MIR structure solution. Acta Crystallogr. Sect. D: Biol. Crystallogr. *55*, 849-861.

Tran, N.T., Den Hengst , C.D., Gomez-Escribano, J.P., and Buttner, M.J. (2011). Identification and characterization of CdgB, a diguanylate cyclase involved in developmental processes in *Streptomyces coelicolor*. J. Bacteriol. *193*, 3100-3108.
